# ARAware: Assisting Visually Impaired People with Real-Time Critical Moving Object Identification

**DOI:** 10.3390/s24134282

**Published:** 2024-07-01

**Authors:** Hadeel Surougi, Cong Zhao, Julie A. McCann

**Affiliations:** 1 Department of Computing, Imperial College London, London SW7 2AZ, UK; j.mccann@imperial.ac.uk; 2 National Engineering Laboratory for Big Data Analytics, Xi’an Jiaotong University, Xi’an 710049, China; congzhao@xjtu.edu.cn

**Keywords:** moving object identification, deep learning, collision prediction, risk classification

## Abstract

Autonomous outdoor moving objects like cars, motorcycles, bicycles, and pedestrians present different risks to the safety of Visually Impaired People (VIPs). Consequently, many camera-based VIP mobility assistive solutions have resulted. However, they fail to guarantee VIP safety in practice, i.e., they cannot effectively prevent collisions with more dangerous threats moving at higher speeds, namely, Critical Moving Objects (CMOs). This paper presents the first practical camera-based VIP mobility assistant scheme, ARAware, that effectively identifies CMOs in real-time to give the VIP more time to avoid danger through simultaneously addressing CMO identification, CMO risk level evaluation and classification, and prioritised CMO warning notification. Experimental results based on our real-world prototype demonstrate that ARAware accurately identifies CMOs (with 97.26% mAR and 88.20% mAP) in real-time (with a 32 fps processing speed for 30 fps incoming video). It precisely classifies CMOs according to their risk levels (with 100% mAR and 91.69% mAP), and warns in a timely manner about high-risk CMOs while effectively reducing false alarms by postponing the warning of low-risk CMOs. Compared to the closest state-of-the-art approach, DEEP-SEE, ARAware achieves significantly higher CMO identification accuracy (by 42.62% in mAR and 10.88% in mAP), with a 93% faster end-to-end processing speed.

## 1. Introduction

Visually Impaired People (VIPs) face a variety of daily challenges due to their impaired visual capability to perceive their surroundings. One significant challenge is collision-free mobility in outdoor environments since various autonomous moving objects such as cars, motorcycles, bicycles, and pedestrians are present. These objects have different properties, i.e., different sizes, varying moving patterns and speeds, and varied approaching times to a VIP, and this brings various potential risks to the physical safety of VIPs or their likely death [1]. For example, cars are more hazardous than bicycles [2], and compared to slow cars (e.g., <15 km/h), fast cars (e.g., >50 km/h) pose a more significant potential of death and arrive at a much shorter time [3]. Effectively detecting such threats is a critical challenge shared by other fields, such as autonomous cars [4] and robotics [5], emphasising the importance of enhancing safety. According to [6], in India, more than 30,000 VIPs are killed annually due to mobility troubles, and about 400 VIPs are hospitalised as the result of pedestrian–vehicle crashes. This demonstrates the significance of detecting such ambient threats, considering the aforementioned properties, to VIPs’ safety and autonomy in their daily mobility. Generally, VIP mobility assistance approaches are divided into conventional tools, e.g., white canes and guide dogs, and technological aids, i.e., non-vision-based (e.g., Smart Cane [7]) and vision-based (e.g., Bbeep [8]) solutions [9]. Compared with traditional tools, technological solutions provide more advantages in terms of detection range, with the potential to provide a more full and precise perception of threats without the need to train animals [10,11]. Providing VIPs with enough information (i.e., spatial and contextual) about surroundings threats can significantly improve their mobility [12].

To capture richer information, current research is focusing on vision-based solutions for VIP mobility assistance. Considerable existing works have successfully aided VIPs in avoiding obstacles using different kinds of cameras (e.g., stereo [8,13], RGB [14,15], and RGB-D [16]). However, such works significantly fall short of being able to identify more serious threats (e.g., faster-moving cars). This is due to a practical deficiency in their object identification methods (e.g., lack of object tracking [14]), a very limited detection area (e.g., only up to 10 m [8,14,15,16]), or failure to operate in real-time (e.g., 5 s delay in system’s response time [13]). For example, in [14], a smartphone-based VIP navigation system is introduced to detect static/moving obstacles with information like estimated distance and approximated obstacle approach direction (i.e., front, right, or left). Based on a Convolutional Neural Network (CNN) classifier, this system can switch between fast and stable modes to balance detection accuracy and processing speed. However, ref. [14] cannot accurately predict potential VIP–object collisions due to the lack of tracking object movement. Object tracking is crucial to obtain necessary information (e.g., object velocity and direction) and to construct object movement trajectory, thus predicting collision effectively. The system also has a practical limitation in estimating object–VIP distance, as only 10 m can be roughly approximated. This distance is not sufficient to ensure early warning of high-speed moving objects. Further, its recognition rate is relatively low (i.e., 55%), which increases to 60% when the authors exclude some obstacle types (e.g., potted plants).

In [15], a CNN-based VIP navigation assistant, DEEP-SEE, is proposed, which jointly detects, tracks, and recognises objects encountered by the VIP in urban environments. It detects threats (i.e., cars, motorbikes, bicycles, pedestrians, and obstructions) based on their relative position to the VIP, and warns about these threats with different priorities (e.g., cars have the highest priority). However, DEEP-SEE can only identify potential collisions up to a distance of approximately 6 m, with an average collision time of only 1∼2 s, even for slowly moving objects (e.g., <22 km/h). Generally, such a short time available for VIPs to react is insufficient. Moreover, because DEEP-SEE does not distinguish between objects moving at different speeds, the suggested fixed 6 m safety area (i.e., the trapezium of interest) fails to protect VIPs from fast-moving objects (e.g., cars and motorbikes). For instance, if a vehicle approaches at 35 km/h within this fixed zone, the VIP would only have 0.62 s to respond, which is far too short. For VIP safety, the safety zone must be adjusted based on the speed of oncoming objects, providing ample time to avoid such threats. Finally, the threat priority mechanism in DEEP-SEE is not comprehensive, for example, a fast-moving low threat object far away may collide with the VIP before a slow-moving high threat object that is quite close to the VIP. In addition, being able to process 20 frames in 1 s (i.e., with a 20 fps processing speed on an NVIDIA GTX 1050 GPU), DEEP-SEE cannot process the 30 fps input video in real-time (on the equivalent hardware).

According to [17], real-time video processing is considered to be the ability to immediately process all frames captured, e.g., if a camera obtains 30 frames per second, the processing time of each frame should be ≤0.033 s (33 ms). The notion of ‘real-time’ is an essential requirement to prevent losing critical information (e.g., spatial, temporal and semantic information) from the input video frames, which is necessary for accurate and in-time collision prediction. Based on specific application needs, a frame rate of 10 fps or more may be adequate for detecting fast-moving objects, whereas a lower frame rate of 2∼3 fps could suffice for slow-moving objects [18]. However, an object detection rate lag behind the camera’s capture rate creates accumulating delays, significantly impacting real-time performance [18]. Studies on VIP assistance [19,20] recommend a minimum frame rate of 30 fps to ensure real-time performance.

In [8], a stereo camera-based system, Bbeep, is proposed to assist VIP mobility in crowded environments like airports by alerting not only the VIP but also nearby pedestrians to clear the path of the VIP. It tracks the movement of sighted pedestrians and predicts potential VIP–pedestrian collisions in real-time. Based on depth data from a stereo camera, Bbeep predicts the future trajectories of sighted pedestrians and will trigger audible alarms if a pedestrian is predicted to be located within a 1 m-wide safety path with the VIP. When potential collisions are identified, three types of warning signals can be emitted, i.e., a low-urgency beep (collision within 5 s), an intermediate-urgency beep (collision within 2.5 s), and a stop sound (imminent risk within 70 cm). However, Bbeep is designed for pedestrians, and it cannot be extended to predict the position of other objects like cars with higher moving speeds or objects with less predictable moving patterns. Further, its limited ‘safety zone’ and passive warning signal emission method (i.e., simple beeps alarming nearby pedestrians to actively avoid colliding with the VIP) cannot effectively prevent the VIP from colliding with more critical objects like cars, motorbikes, and bicycles (that are more difficult to both obtain and react to the beeping alarms).

In summary, the state-of-the-art camera-based VIP mobility assistant solutions are promising but cannot effectively prevent collisions between a VIP and different types of dangerous objects moving at low-to-high speeds (i.e., Critical Moving Objects, CMOs). A more practical VIP assistance scheme should identify and warn the VIP about such threats efficiently and provide enough time for the VIP to safely react, i.e., avoid CMO in time. Further, this CMO identification and warning scheme should be conducted in real-time from the end-to-end (E2E), i.e., from capturing a scene to triggering a warning, without missing critical information in the input video’s frames.

In this paper, we present ARAware (or the Ambient Risk Awareness) assistant scheme to help the VIP to avoid different classes of CMOs moving at varying speeds in outdoor environments. As far as we are aware, ARAware is the first practical camera-based VIP mobility assistant scheme that effectively and efficiently identifies challenging CMOs and provides longer VIP reaction times for CMO avoidance by addressing the combined issues of real-time CMO identification, risk level-based CMO classification, and prioritised CMO warning simultaneously; to bring these together effectively requires different thinking and novel algorithms. This is to overcome the limitations of current VIP mobility assistant solutions and ensure VIP safety. The contributions of this paper can be summarised as follows:

- We provide an E2E camera-based CMO avoidance pipeline from video capture to message delivery, where the essential properties of the perception of the VIP in practice are comprehensively considered. Particularly, we design a deep learning-based moving object tracking capable of achieving real-time processing without missing critical information in the input video. Additionally, we develop a novel object–VIP collision prediction method based on the object’s moving direction for effective collision prediction. We also construct a 2D to 3D coordinate projection method for more accurate object–VIP distance estimation, which significantly improves the performance of object–VIP collision prediction, and thus CMO identification. The ARAware scheme is customizable and designed for future-proofing; according to the requirements of the VIP, other object detectors, trackers, and distance estimators can be integrated for higher accuracy or faster processing speed.
- We provide a systematic CMO risk level estimation scheme, derived from comprehensive empirical studies, that provides an indicator of the damage caused to the VIP via collisions of different types of objects moving at different speeds in practical settings. We classify the identified CMOs with different risk levels, and develop a novel prioritised CMO warning strategy, based on the CMOs risk levels and predicted collision times, to immediately warn about high-risk threats for the safety of the VIP, and to postpone the warning of low-risk threats until an acceptable deadline (sufficient for VIP to react) to reduce potential false alarms.
- We implement a real-world prototype of ARAware using commercially available devices (i.e., an RGB camera, a laptop, and bone conduction headphones), and validate its effectiveness and efficiency based on extensive real-world field trials. Experimental results demonstrate that, consuming 30 fps input video, ARAware manages to accurately identify CMOs with 97.26% overall mean Average Recall (mAR) and 88.20% overall mean Average Precision (mAP), precisely classify their risk levels with 100% overall mAR and 91.69% overall mAP, and achieve real-time processing with an average E2E processing speed of 32 fps. Furthermore, the ARAware warning emission strategy manages to effectively alert the VIP about identified CMOs with different risk levels, where high-risks are alerted immediately (e.g., cars moving at 60 km/h are alerted at least 6 s in advance), and, low-risks are not alerted until the deadline is reached (e.g., pedestrians moving at 3 km/h are alerted 5 s ahead, instead of 15.21 s in advance when they are first identified), which effectively reduces potential false alarms, considering that the accuracy of detecting proximate objects is higher than that of distant objects.

The core novelty of this paper lies in seamlessly integrating the state-of-the-art models (YOLOv8, Deep SORT, DisNet) with our novel developed algorithms (collision prediction, risk level estimation, and risk-aware prioritised CMO warning) to build a unified, real-time scheme that accurately identifies different classes of CMOs moving at low-to-high speeds, providing a more comprehensive and practical solution for VIP safe mobility. This integration, which combines many different techniques while effectively achieving real-time and accuracy requirements, was a significant challenge we successfully addressed.

The rest of the paper is organised as follows. In Section 2, we discuss the related work. The schematic design of ARAware is presented in Section 3. Section 4 presents the ARAware prototype implementation and the methodology used for evaluation. Section 5 shows and analyses the evaluation results. We discuss the limitations of ARAware in Section 6. Section 7 concludes this work.

## 2. Related Work

In this section, we discuss the existing studies of camera-based VIP mobility assistant systems. Typically, such systems depend on computer vision-based algorithms (e.g., CNN-based classifiers) applied to images and videos collected by different types of cameras (e.g., stereo [13], RGB [21,22], and RGB-D [23,24]).

The Navigation Assistance for Visually Impaired (NAVI) system is proposed in [23], where a low-cost RGB-D camera is used. Here, the range information, collected by a built-in infrared-based depth sensor, and visual information are fused for major structural element detection (i.e., walls and floors). Although the system manages to accurately conduct range-based floor segmentation (with 99% precision), it only works for static structural obstacles in indoor environments. In [24], a specific CNN, KrNet, is proposed as a road barrier recognizer to help VIP in outdoor environments by perceiving the entrance and exit of construction areas and car parks. Here, a smart glass with an RGB-D sensor is used to capture images with depth information. For a specific image, when KrNet detects barriers within 20 m, the VIP will be notified through a headphone and guided to the destination. Running on resource-limited mobile devices, KrNet, however, can only detect a single class of objects.

In [21], an obstacle detection method, Deformable Grid (DG), is proposed for VIP collision prevention. DG is a grid that can be gradually deformed according to object movements in the scene and returns to its regular shape when no object exists. Such a method is integrated with a glasses-type wearable device with an RGB camera and a Bluetooth headphone, where the risk of collision is detected based on the extent of deformation; a corresponding audio feedback is generated. In [22], a vertex deformation function is used to improve the DG accuracy and processing speed. However, such methods can neither recognize the class of obstacles nor identify their risk types. They only emit a signal to indicate incoming objects, i.e., temporal frequency becoming higher when the obstacle gets closer to the VIP, leading to false alarms which unnerve the VIP needlessly. In [25], a deep learning-based object recognition system is proposed to guide the VIP in outdoor environments. Here, video frames are captured by a smartphone and then uploaded to a CNN-based object recognizer on the cloud, and the information (i.e., object class) is conveyed to the headphone of the VIP. The system can recognize 11 classes of common objects like cars, motorbikes, bicycles, and pedestrians with a 96.4% recognition rate. However, it can only classify obstacles, and no depth information of detected objects is provided; thus, potential collisions threatening the safety of the VIP cannot be predicted.

In [26], a smartphone-based obstacle detection and classification system is proposed for VIP mobility assistance in both indoor and outdoor environments. The system extracts points of interest in the captured frames using the Scale Invariant Feature Transformation (SIFT) and the Speed-Up Robust Feature Extractor (SURF) methods. The extracted points are tracked using the Lucas–Kanade algorithm, and classified as vehicles, bicycles, pedestrians, and obstacles using the Support Vector Machine (SVM) and the Bag of Visual Words (BoVW) models. Two levels of obstacle risk are provided, i.e., urgent and normal, which are determined based on the obstacle’s position and moving direction (approaching or departing from the camera focus). However, due to the high complexity, this system achieves a limited processing speed (i.e., 7 fps on the equivalent hardware). When processing general video clips with a frame rate of 30 fps, this method misses considerable numbers of important frames, which may threaten the safety of the VIP. For example, if a car approaches the VIP at 54 km/h (15 m/s), it would cover 15 m in a second. With only a 7 fps detection rate, the system might miss the critical frames depicting the car’s close proximity to the VIP due to frame gaps. This delay in detection significantly reduces the reaction time of the VIP to avoid the potential hazard.

In [13], a novel stereo-based obstacle avoidance method is proposed to enhance VIP safety. It uses a custom lightweight YOLOv5 model to detect obstacles and employs an adaptable grid-based approach to prioritise the dangerous obstacles based on their proximity to the VIP (the closer is the most danger). However, prioritising objects solely based on proximity cannot always guarantee VIP safety. Factors like object type and speed can pose a more significant threat than distance alone. Moreover, the system’s stereo-based distance estimation accuracy diminishes as the distance from the camera to obstacles increases, and collecting stereo images requires complex alignment and calibration processes [27,28]. The system also exhibits a 5 s delay between obstacle detection and the VIP alert, potentially being a safety hazard, especially with fast-moving objects.

In [29], a method is proposed for the real-time detection of the presence of moving objects along with their trajectory (i.e., upward, downward, forward, backward, left-to-right, and right-to-left). However, the system is limited to identifying the moving direction only and cannot recognize the class of moving objects, hindering the effectiveness of predicting potential object–VIP collisions. In [30], a simple mobile application for real-time object identification is proposed. Such an application helps the VIP to perceive surrounding objects by capturing photos. Based on the photos, the application notifies the VIP about the class and the absolute location of the identified objects. However, the application does not collect the object’s depth information and thus cannot predict potential collisions.

In [31], a smartphone-based obstacle detection system enhances the outdoor mobility of VIPs using deep learning. It employs a DeepLabV3 model for real-time object identification and uses triangulation for estimating VIP–object distances. Offering “stable” and “fast” modes, it prioritises accuracy with detailed voice guidance in unfamiliar environments and prioritises speed with real-time object detection in crowded areas. Voice feedback informs VIPs about obstacle types, directions, and distances. However, the system’s triangulation-based distance estimation is inaccurate and highly error prone [32,33], especially for large distances, as small triangulation errors can lead to significant distance errors [34]. In [35], a portable real-time system aids VIPs in perceiving nearby objects and pedestrians. It uses a YOLOv3 model with a single camera mounted on a Raspberry Pi board for object detecion. While excelling at VIP–pedestrian distance estimation (98.8% accuracy), its range is limited to 10 m and exclusively tailored to pedestrians. This mean it may not accurately estimate distances for other objects (e.g., cars) or beyond the specified limit.

## 3. ARAware Schematic Design

**Figure 1:**
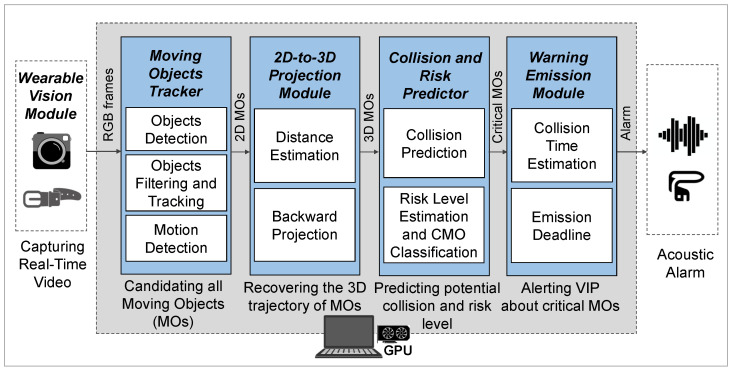
The general schematic architecture of ARAware.

In this section, we present the design of the ARAware scheme. The general schematic architecture of ARAware is illustrated in Figure 1, which comprises **five** major modules, i.e., the Wearable Vision Module (WVM) for real-time video capture, the Moving Object Tracker (MOT) that detects and tracks moving objects from the video frames provided by the WVM, the 2D-to-3D Projection Module (PM) that converts the 2D pixel position of the moving objects in video frames into a 3D metric position in the Camera Coordinate System (CCS, explained later in Section 3.3), the Collision and Risk Predictor (CRP) that identifies the Critical Moving Objects (CMOs) that may collide with the VIP among objects tracked by the MOT, and therefore estimating the risk level of each CMO, and the Warning Emission Module (WEM) that acoustically alerts the VIP about CMOs. Details of each module are presented as follows.

### 3.1. The Wearable Vision Module

In the WVM, an RGB camera with a fixed focal length is used for video capture. To avoid potential jarring and disorientation caused by natural head movements and walking-induced camera shaking, we mounted the camera on a stabilisation stick attached to the chest harness of the VIP while enabling the camera to be elevated to the eye level of the VIP to provide a human-like field of view. The height of the camera depends on the height of the VIP. The camera captures video at a specific frame rate (i.e., the number of video frames captured in 1 s) with a specific resolution, and transmits all captured frames to the MOT through a USB cable.

### 3.2. The Moving Object Tracker

The MOT aims to detect and track targeted classes of moving objects in video frames from the WVM, which is conducted in three steps, i.e., object detection, object filtering and tracking, and motion detection.

#### 3.2.1. Object Detection

To allow the VIP to react in a timely manner to potential collisions, real-time and accurate object detection is paramount. To achieve this, we use a CNN-based object detector, YOLOv8, a recent version of the well-established YOLO object detection models. By integrating an optimised CSPDarknet53 backbone, an anchor-free detection, and a novel loss function, Yolov8 achieves superior performance in both detection accuracy and processing speed (end-to-end (E2E) inference time) within the YOLO series [36,37]. Considering its prominent and stable performance on different public datasets (e.g., COCO [37] and CrowdHuman [38]), we use the YOLOv8 model pre-trained on the COCO dataset (a large-scale object detection benchmark dataset) as our object detector. For each captured video frame, with a specific resolution, the object detector localises and classifies all objects in the frame. The output is a list of bounding boxes of detected objects, each associated with an object class label and detection confidence score indicating the likelihood that the box contains an object [37].

#### 3.2.2. Object Filtering and Tracking

According to [37], our pre-trained YOLOv8-based object detector manages to detect 80 different classes of objects. Since we concentrate on outdoor moving objects threatening the safety of the VIP, a class-based object filtering process is conducted to extract targeted objects including cars, motorbikes, bicycles, and pedestrians. Note that any other class of objects can be included according to the VIP requirements without the need to train the detector.

Since the object detector provides independent bounding boxes of different detected objects, and the bounding boxes of the same object in different frames are not correlated, associating the detected objects across frames is necessary to estimate object movement and its threat to the VIP. To achieve this, we use the Deep Simple Online Real-Time (Deep SORT) model pre-trained on MOT16 [39] as our object tracker for real-time multi-object tracking considering its fast processing speed and competitive accuracy. Receiving bounding boxes of all targeted objects (within all frames where object detection is conducted), Deep SORT assigns a unique ID to each object, and sorts all bounding boxes of the object temporally to form a trajectory in an online manner.

**Figure 2:**
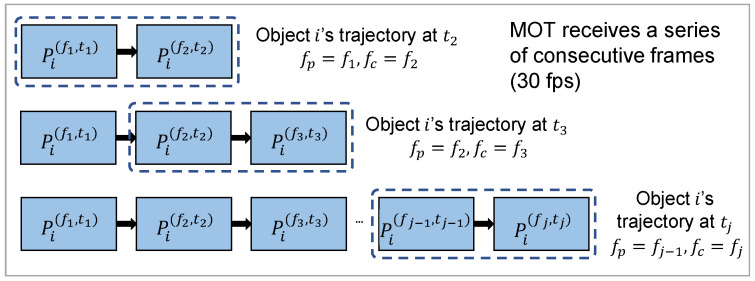
Moving object trajectory update. Note: When the trajectory is updated at $t_{j}$, the estimated distance and estimated moving speed are also updated to calculate the latest expected collision time.

One thing should be noted is that, to improve the processing speed of ARAware, in the trajectory of a specific object, we only record sorted bounding boxes of the latest two detected frames, i.e., the previous frame $f_{p}$ detected at time $t_{p}$, and the current frame $f_{c}$ detected at time $t_{c}$. Recording only the latest two bounding boxes helps reduce computational demands and memory use. This maintains real-time processing while keeping sufficient up-to-date data for accurate VIP–object collision prediction (detailed in Section 3.4.1). The online update process of the object trajectory is illustrated in Figure 2.

#### 3.2.3. Motion Detection

For computational simplicity, we develop a straightforward motion detector to identify targeted objects that are moving, which can be used when the VIP (camera) is still or moving. Note that this detector is also necessary to separate the camera motion from an object’s motion to accurately estimate the object’s speed (explained later in Section 3.4.1). First, we use the Oriented FAST and Rotated BRIEF (ORB) feature detector [40] to estimate the camera motion by finding the same features within two consecutive frames, chosen because it outperforms counterparts like SIFT and SURF in terms of computation speed and costs. Among all detected features, our motion detector selects the best 25∼50 matches for the trade-off between feature utilisation maximisation and computational cost reduction. Obtaining immobile features (i.e., features extracted from static structures like buildings) is essential for effective camera motion estimation. Therefore, our detector excludes features located within the bounding boxes of the targeted detected moving objects. Then, for each feature $M_{i}$, its 2D pixel positions $M_{i}^{\left(f_{p},t_{p}\right)}={\left(x_{m},y_{m}\right)}_{i}^{\left(f_{p},t_{p}\right)}$ at $f_{p}$ and $M_{i}^{\left(f_{c},t_{c}\right)}={\left(x_{m},y_{m}\right)}_{i}^{\left(f_{c},t_{c}\right)}$ at $f_{c}$ are compared. If such pairs of positions of the majority of features (i.e., ≥50%) are identical, the camera is identified as static, and otherwise as moving. The reason is that even if we exclude the extracted features of targeted moving objects (as mentioned above), some features are likely to be extracted from non-targeted moving objects like pets and immobile structures like trees with moving leaves.

Then, for each targeted object $o_{i}$, its bounding box is defined as ${\left(x_{1},y_{1},x_{2},y_{2}\right)}_{i}$, i.e., the minimum and maximum, 2D pixel coordinates of the rectangular border that fully encloses the detected object in the video frame. By receiving ${\left(x_{1},y_{1},x_{2},y_{2}\right)}_{i}^{\left(f_{p},t_{p}\right)}$ and ${\left(x_{1},y_{1},x_{2},y_{2}\right)}_{i}^{\left(f_{c},t_{c}\right)}$ in object $o_{i}$’s trajectory, the motion detector calculates the central position of $o_{i}$ at $f_{p}$ and $f_{c}$ as $P_{i}^{\left(f_{p},t_{p}\right)}={\left(x,y\right)}_{i}^{\left(f_{p},t_{p}\right)}$ and $P_{i}^{\left(f_{c},t_{c}\right)}={\left(x,y\right)}_{i}^{\left(f_{c},t_{c}\right)}$, respectively. For the static camera, if the Euclidean distance between the $P_{i}^{\left(f_{p},t_{p}\right)}$ and $P_{i}^{\left(f_{c},t_{c}\right)}$ is greater than ϵ (the ϵ can be set as any small value to tolerate computational errors [41]; in our experiments, we set ϵ as zero for stricter object motion detection, as the safety of the VIPs is our fundamental goal), $o_{i}$ is identified as a moving object, and its trajectory is passed to CRP. For moving camera, the detector calculates the displacement between the $M_{i}^{\left(f_{p},t_{p}\right)}$ and $M_{i}^{\left(f_{c},t_{c}\right)}$ of the best matched $M_{i}$, at $f_{p}$ and $f_{c}$, respectively, which is defined as $D_{i}^{M_{p},M_{c}}={\left(x_{m}^{t_{c}}-x_{m}^{t_{p}},y_{m}^{t_{c}}-y_{m}^{t_{p}}\right)}_{i}$; then, if $P_{i}^{\left(f_{c},t_{c}\right)}-D_{i}^{M_{p},M_{c}}\neq P_{i}^{\left(f_{p},t_{p}\right)}\pm \epsilon$, $o_{i}$ is identified as a moving object, and its trajectory is passed to CRP. The $D_{i}^{M_{p},M_{c}}$ will be used later in Equations (12) to (16) for camera motion separation.

### 3.3. The 2D-to-3D Projection Module

**Figure 3:**
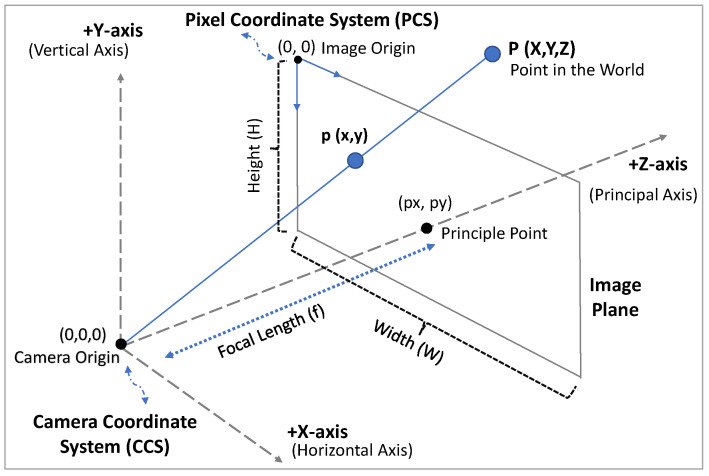
Demonstration of the reference Camera Coordinate System. Note: The camera centre is at the origin (0, 0, 0), the X-axis and Y-axis are the horizontal and vertical axes that are parallel to the image plane axes, and the Z-axis is the principal axis that is orthogonal to the image plane.

For all targeted moving objects tracked by MOT, their 2D trajectories in the captured video frames are transmitted to PM. However, real-world trajectories of moving objects are necessary for the accurate prediction of potential object–VIP collisions. To achieve this, PM first estimates the real-world distance between the VIP and the moving objects, then transforms the objects’ 2D trajectories in the Pixel Coordinate System (PCS) to 3D trajectories in the Camera Coordinate System (CCS). The CCS is a real-world 3D coordinate system with the camera as the origin (see Figure 3) and measurement units in m.

#### 3.3.1. Distance Estimation

To successfully recover an object’s 3D metric position from its 2D pixel position, one needs to obtain the distance from the camera to the object. Further, a precise and *long-range* distance estimation method contributes to more accurate and earlier collision predictions for guaranteeing VIP safety. To achieve this, we employ the pre-trained DisNet model [42] (trained based on the YOLO resulting bounding boxes [43]) over other deep learning-based methods, as it is a simple, fast, and precise long-range monocular camera-based distance estimation model. Unlike classical methods, the accuracy of DisNet is not affected by how far the object is from the camera, and it requires no prior knowledge about either the scene or the camera parameters.

DisNet is a multi-layer neural network developed for autonomous long-range obstacle detection in railway applications. It consists of a six-dimensional input layer (feature vector), three hidden layers, each with 100 neurons, and a one-dimensional output layer (distance in m). For the 2D bounding box of each detected object, the input feature vector is calculated as follows:

(1) $$V_{f}=\left[\frac{1}{R_{h}},\frac{1}{R_{w}},\frac{1}{R_{d}},C_{h},C_{w},C_{d}\right],$$

where,

(2) $$R_{h}=\frac{BB_{h}}{I_{h}},R_{w}=\frac{BB_{w}}{I_{w}},R_{d}=\frac{BB_{d}}{I_{d}},$$

where $R_{h}$, $R_{w}$ and $R_{d}$ are the ratios of the object’s bounding box dimensions (i.e., height, width, and diagonal) to image dimensions in pixels, respectively. $C_{h}$, $C_{w}$ and $C_{d}$ denote the predefined values of average dimensions (i.e., height, width, and depth) of an object of a particular class in cm. Inspired by [42], predefined dimensions of our targeted classes are set as follows:

- Cars: $C_{h}$ = 160 cm, $C_{w}$ = 170 cm, and $C_{d}$ = 400 cm;
- Motorbikes: $C_{h}$ = 124 cm, $C_{w}$ = 65 cm, and $C_{d}$ = 190 cm;
- Bicycles: $C_{h}$ = 105 cm, $C_{w}$ = 40 cm, and $C_{d}$ = 175 cm;
- Persons: $C_{h}$ = 175 cm, $C_{w}$ = 55 cm, and $C_{d}$ = 30 cm.

DisNet then receives the feature vector $V_{f}$ and generates the distance $Z^{t}$ (in m) from the camera (i.e., the VIP) to the detected object at time *t* in real-time:

(3) $$Z^{t}=DisNet\left(V_{f}\right).$$

Note that when an object (e.g., motorbike) is far away and has properties similar to another object (e.g., bicycle), the YOLOv8-based object detector may misclassify the object; therefore, the distance estimate is affected (i.e., estimation error becomes larger) since it strongly depends on the object’s class dimension. However, this does not affect our work because when the object is far away, its dimensions on the image are small; in addition, the difference in the actual dimensions of similar objects (bicycle and motobike) is generally small (15∼25 cm based on the above predefined class dimensions), resulting in a negligible estimation error rate. Even if there is an initial misclassification, it will be corrected as the object gets closer. Further, when the correctly classified object (initially moving towards the VIP) changes its moving direction (turning left or right), the size of the bounding box surrounding the object increases; thus, the error rate increases (it appears closer). However, this also does not affect our work because if the object changes its direction, this means no object–VIP collision; thus, we ignore it.

#### 3.3.2. Backward Transformation

In general perspective projections, defined as the process of converting 3D scenes into 2D images, the real-world distance and angle are not preserved from a scene [44]. To achieve accurate distance and moving angle approximations for collision prediction (explained more in the next section), we perform a backward transformation to retrieve the 3D object’s position in the CCS. Based on the Pinhole Model [45] and given the distance $Z^{t}$ in Equation (3), as shown in Figure 3, the 2D object’s position in the PCS is transformed into a 3D position in the CCS as follows:

(4) XOtC=(xt−px)Ztfx,

(5) YOtC=(yt−py)Ztfy,

(6) ZOtC=Zt,

where $\left(x^{t},y^{t}\right)$ is the 2D pixel position of an object at time *t*, $\left(p_{x},p_{y}\right)$ is the principal point of the camera in pixels, and $f_{x}$ and $f_{y}$ are the X-axis and Y-axis of the camera’s focal length components in pixels. Here, both the principal point and the focal length are the intrinsic parameters of a camera which can be estimated using the camera calibration based on OpenCV 4.1 (https://docs.opencv.org/master/dc/dbb/tutorial_py_calibration.html (accessed on 2 April 2024)). Here, PM transforms all targeted moving objects’ 2D trajectories in the PCS into 3D trajectories in the CCS.

### 3.4. The Collision and Risk Predictor

On receiving 3D trajectories from the PM, the CRP first predicts potential collisions by analysing object movements, then classifies critical objects according to their risk levels.

#### 3.4.1. Collision Prediction

We develop a method based on vector calculus [46] to identify objects that may collide with the VIP, i.e., Critical Moving Objects (CMOs). To prevent heavy computation for complex projections in a real-world coordinate system with a fixed origin, we use the CCS, whose origin is always the current position of the VIP, as the reference coordinate system (see Figure 3).

**Figure 4:**
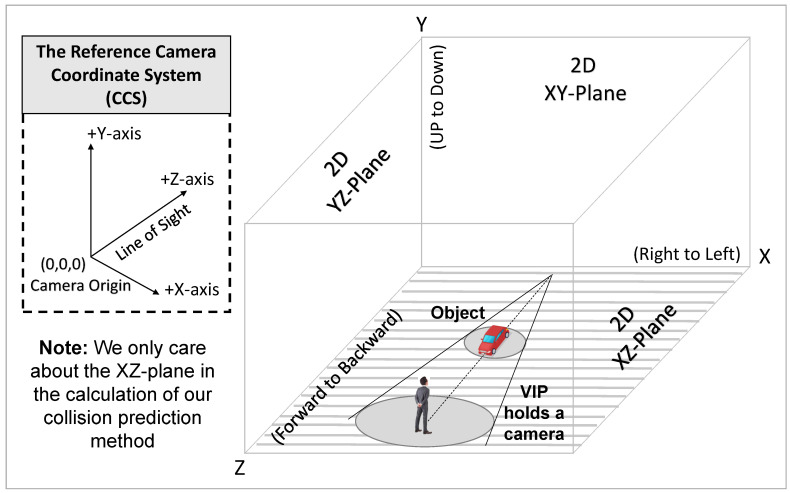
Demonstration of the 2D XZ-plane considered in collision prediction. Note: The 2D XZ-plane is the ground plane shared by VIP and the detected object.

**Figure 5:**
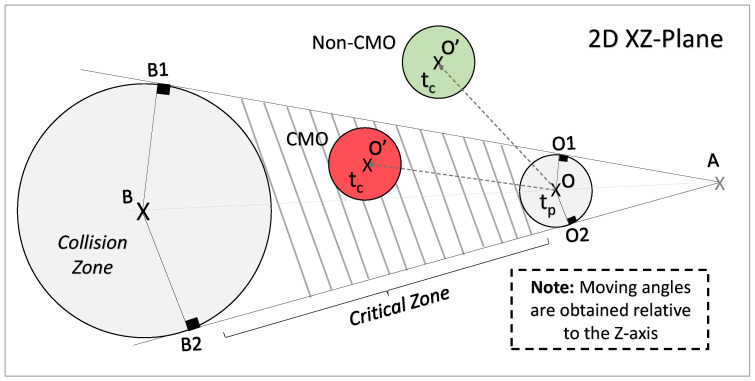
Critical zone estimation based on geometric principles.

As shown in Figure 4 and Figure 5, we assume that the VIP is at point *B*, which is the centre of the VIP’s circular collision zone with a radius of $r_{B}$ on the 2D XZ-plane (i.e., the ground plane). We assume that an object occupies a circular area with a centre at point *O* and a radius of $r_{O}$ at $t_{p}$, which moves to point $O^{\prime }$ at $t_{c}$. Note that we assume $r_{B}\gg r_{O}$ for the safety consideration (i.e., the VIP’s collision zone should be large enough to detect all possible threats). For simplicity, we assume that the VIP (camera) is static, and any object can move at a velocity of v=|v|∠θ, where |v| is the speed, and ∠θ is the moving angle (i.e., the angle between the object moving direction and the Z-axis of the CCS).

**CMO Identification:** As shown in Figure 5, we define the critical zone with two boundaries, i.e., $\overset{↔}{B_{1}O_{1}}$ and $\overset{↔}{B_{2}O_{2}}$, which are two common tangents of circles $C_{B}$ and $C_{O}$ intersected at point *A*. Then, we define three angles as follows:

- The angle ∠α between $\bar{AB}$ and $\bar{AB_{1}}$ (see Appendix A for the derivations), which can be expressed as:
  (7) $$\alpha =tan^{-1}\left(\frac{1}{\sqrt{\frac{d^{2}}{{\left(r_{B}-r_{O}\right)}^{2}}-1}}\right),$$
  where $d=\;|\bar{OB}|$ is the Euclidean distance between the VIP and the object at $t_{p}$, which is calculated using Equations (4) and (6) as follows:
  (8) d=(XBtpC−XOtpC)2+(ZBtpC−ZOtpC)2,
  where XBtpC and ZBtpC are the X-axis and Z-axis coordinates of the VIP point *B*, while XOtpC and ZOtpC are the X-axis and Z-axis coordinates of the object point *O* at $t_{p}$. We ignore the Y-axis coordinates (i.e., YBtC=0 and YOtC=0) since we assume that the camera motion at the Y-axis is negligible, as it is vertical to the ground plane.
- The angle ∠ϕ of the displacement vector from *O* to *B*, which is expressed as:
  (9) ϕ=tan−1XBtpC−XOtpCZBtpC−ZOtpC,
- The angle ∠β of the displacement vector from *O* to $O^{\prime }$, which can be calculated as:
  (10) β=tan−1XOtcC−XOtpCZOtcC−ZOtpC,

With angles α, β, and ϕ, if ϕ−α≤β≤ϕ+α, the object is identified as a CMO since it is moving within the critical zone and may threaten the VIP. Otherwise, it is a non-critical object. Note that the CMO identification is based on the moving angle (i.e., direction), not the location of the object. For instance, the moving object coloured with green in Figure 5 will not be classified as a CMO since it is moving out of the critical area (i.e., it does not move towards the VIP).

To set up the radius $r_{O}$, we consider that, for the trade-off between the safety of the VIP and scheme usability, the circular zone should be large enough to cover the object’s width in a particular class and at the same time not be much larger than the object’s width to reduce the false alarms. To set up the radius $r_{B}$, we consider that the collision zone should be considerably larger than the object’s zone to ensure the detection of all possible CMO threats to the safety of the VIP. To determine the proper radius setups considering our concerns above, we conducted empirical studies based on extensive real-world experiments. These experiments involved measuring the actual width of objects belonging to our target classes and determining the corresponding radius for a VIP circular zone that would ensure the safety of the VIP. Using these measurements, we marked out critical zones (cones) for each class on the ground and then collected and analysed data on object movement both inside and outside these zones. Based on our analysis, the corresponding radius setups (can be adjusted according to the safety requirements of the VIP) are as follows:

- Cars: $r_{O}$ = 0.9 m, $r_{B}$ = 3 m,
- Motorbikes: $r_{O}$ = 0.45 m, $r_{B}$ = 1.75 m,
- Bicycles: $r_{O}$ = 0.35 m, $r_{B}$ = 1.25 m,
- Persons: $r_{O}$ = 0.25 m, $r_{B}$ = 0.75 m.

It should be noted that since the collision prediction depends on the object’s moving direction only, our CMO identification method can be directly applied to the scenario where the VIP is moving without abrupt direction changes and to the scenario where multiple objects are present.

**CMO Speed Estimation with Removing Camera Motion:** For each CMO, we estimate its moving speed as:

(11) $$v^{\prime }=\sqrt{{v_{x}}^{2}+{v_{y}}^{2}+{v_{z}}^{2}},$$

where

(12) vx=(XOtcC−Dcx)−XOtpCtc−tp,

(13) vy=(YOtcC−Dcy)−YOtpCtc−tp,

(14) vz=ZOtcC−ZOtpCtc−tp±Cz,

where

(15) $$D_{c_{x}}=\left(x_{m}^{t_{c}}-x_{m}^{t_{p}}\right)\frac{Z^{t_{c}}}{f_{x}},$$

(16) $$D_{c_{y}}=\left(y_{m}^{t_{c}}-y_{m}^{t_{p}}\right)\frac{Z^{t_{c}}}{f_{y}}.$$

where, $v_{x}$, $v_{y}$ and $v_{z}$ are the CMO velocity components of the X-axis, Y-axis, and Z-axis, respectively. Both (XOtpC,YOtpC,ZOtpC) and (XOtcC,YOtcC,ZOtcC) are the CMO positions at $t_{p}$ and $t_{c}$, respectively. $D_{c_{x}}$ and $D_{c_{y}}$ represent the camera displacement at the X-axis and Y-axis, respectively. $C_{z}$ is the camera’s velocity in the Z-axis.

It should be noted that, removing camera movement helps to obtain an accurate estimation of the actual speed of the CMO. To achieve this, in Equations (12) and (13), we subtract $D_{c_{x}}$ and $D_{c_{y}}$, which are the X-axis and Y-axis components of $D_{i}^{M_{p},M_{c}}$ (see Section 3.2.3) multiplied by the pixel–meter conversion ratio $Z^{t_{c}}/f$, to remove the effect of the camera movement in the X-axis and Y-axis, respectively. Since the movement of the camera in the Z-axis cannot be estimated by the 2D feature-based motion detection (explained in Section 3.2.3), we assume that the VIP walks at a slow velocity (i.e., ≤3 km/h) in the Z-axis. Generally, VIPs walk slower than sighted people, usually moving at an average speed of 5 km/h, and the walking speed often decreases as the complexity of or uncertainty about the surrounding environments increases [47]. Thus, in Equation (14), we add or subtract (±, based on the relative movement directions of the VIP and CMO along the Z-axis, i.e., forward or backward) a constant value $C_{z}$ from the CMO’s Z-axis velocity, where the $C_{z}$ represents the approximate Z-axis velocity of the VIP and can be adjusted according to the walking speed of the VIP.

Then, by keeping a record of $v^{\prime }$, we calculate the average speed of CMO as:

(17) $$v_{avg}^{\prime }=\frac{1}{n}\sum_{i=1}^{n}v_{i}^{\prime },$$

where *n* is the total number of speeds $v_{i}^{\prime }$ in the record. Compared with $v^{\prime }$, considering that the calculation time of $v_{avg}^{\prime }$ is negligible, we use $v_{avg}^{\prime }$ in the CMO collision time prediction (discussed later in Section 3.5) since it is less impacted by outliers of the CMO speed estimation. In fact, either $v^{\prime }$ or $v_{avg}^{\prime }$ can be used considering the VIP requirements; $v^{\prime }$ should be used for a faster warning delivery, and $v_{avg}^{\prime }$ should be used for a more accurate collision time estimation, see Equation (18).

#### 3.4.2. Risk Level Estimation and CMO Classification

For all CMOs identified, considering their threats to the VIP, we classify them into several groups with different risk levels. In general, CMOs with higher risk levels should be processed with higher priorities to avoid more serious damages to the VIP.

For a reasonable definition of risk levels, we conducted an empirical background study of injuries caused by traffic collisions as follows. According to the UK Department for Transport (DfT) [48], the human injury severity in public road accidents can be classified into three levels, i.e., fatal, serious, and slight. In fact, the injury severity is determined by a number of factors related to vehicles, impacted persons, and environments, where the vehicle speed is a fundamental factor [49]. Many studies concentrate on the relationship between the impact speed of vehicles (i.e., the absolute value of relative speed between the vehicle and the impacted person at the collision moment) and the injury severity [50]. It is generally understood that cars cause minor injuries when the impact speed is below 25 km/h, and fatal injuries when the impact speed exceeds 45 km/h [49]. Motorbikes can cause different fatal injuries [51]; however, there is a lack of numerical studies of the relationship between the impact speed and the injury severity for these. Considering that motorbikes can travel at high speeds like cars, we assume that motorbikes’ damages are close to those caused by cars in general. Bicycles may cause secondary post-impact head injures when the impact speed is higher than 35 km/h [52]. Pedestrians usually walk at a low speed [53] and generally cause no serious injury when they collide with others.

**Figure 6:**
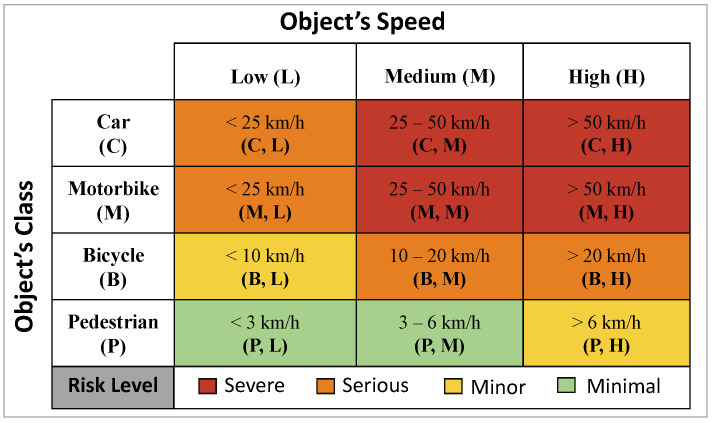
CMO risk level classification based on potential damage.

Based on the above understanding, we use both the type and the moving speed of CMOs as major criteria of risk level classification. Specifically, we define four risk levels of all CMOs, i.e., severe (e.g., fatal injuries), serious (e.g., fractures or injuries cause hospitalisation), minor (e.g., bruises), and minimal (e.g., no injuries or minor scrapes). From [52,53,54], we summarise the general average speed of cars, motorbikes, bicycles, and pedestrians, and set up a mapping between risk levels and CMOs moving at different speeds (see Figure 6). Note that this mapping can be straightforwardly adjusted to include more object classes based on the VIP requirements without the need for further effort (e.g., model training). Such a mapping is used to tag each CMO with a risk level as the input of WEM. One thing should be noted is that, for the safety of the VIP, we set the speed threshold in the mapping as half of the general average speed (i.e., we deliberately assign higher risk levels to CMOs for stricter warning). By risk classification, we aim to improve the efficiency of our scheme through early high-risk threat warnings (i.e., severe and serious risks) and reducing false alarms caused by threats with low risks (i.e., minor and minimal risks). This prioritisation enables the quick and timely reaction of the VIP.

### 3.5. The Warning Emission Module

For CMOs with different risk levels, WEM aims to warn the VIP about potential threats in a timely, efficient, and highly usable manner. Specifically, the following three requirements need to be satisfied:

- In-time warning of CMOs, i.e., $t_{cl}^{\prime }\leq t_{dl}$, see Equations (18) and (19), where enough time should be left for the VIP to react.
- Prioritising warning messages of CMOs with different risk levels (i.e., scaled from 1 to 4, where 1 denotes the highest risk and 4 denotes the lowest risk).
- Reducing false alarms as much as possible to ensure scheme usability.

Considering these requirements, we develop a warning emission method as follows.

**Warning Emission Priority:** We assign the warning emission priority according to the risk level of the CMOs. Specifically, where multiple warning messages for different CMOs occur at the same time, messages of CMOs with higher risk levels will be sent earlier. For CMOs with ‘severe’, ‘serious’, ‘minor’, and ‘minimal’ risk levels, we assign ‘1st’, ‘2nd’, ‘3rd’, and ‘4th’ priorities, respectively. For CMOs with the same priority, their warning messages are emitted according to the expected collision time, i.e., the shortest first.

**Trade-off between In-time Warning Communication and False Alarm Reduction:** On the one hand, for in-time warning emission, it is intuitive to send warning messages as soon as the CMOs are identified. However, due to the property of our object detector and tracker, CMO identifications tend to be less accurate using early frames, where CMOs are just identified, and objects are generally far away from the camera. Correspondingly, the accuracy of collision and risk prediction will also be downgraded. Moving objects identified as critical at first may change direction before actually hitting the VIP. Considering these factors, immediate warning emission may cause considerable false alarms and hinder the scheme usability. On the other hand, to ensure that the VIP has enough time to avoid predicted collisions, it is necessary to emit early warnings.

For the trade-off between in-time warning and false alarm reduction, for each CMO, we determine its warning emission time based on both the estimated collision time and the warning emission deadline, instead of the object–VIP distance adopted by the existing warning emission methods [8,15]. The reason is that in the distance-based method, warning messages of objects with the same distance to the VIP are emitted at the same time. However, if these objects are moving at different speeds, they may collide with the VIP at different time points, which results in severe threats to the safety of the VIP.

Specifically, as shown in Figure 5, once a CMO is identified, we obtain its object–VIP distance estimation ZOtC according to Equations (3) and (6), and the average speed estimation $v_{avg}^{\prime }$ according to Equation (17). Therefore, the CMO collision time is estimated as:

(18) tcl′=ZOtCvavg′+vVIP′,

where $v_{VIP}^{\prime }=C_{z}$, the approximate velocity of the VIP as mentioned in Section 3.4.1. Note that, for any CMO identified, its estimated collision time is updated whenever its trajectory is updated. Then, we set up different warning emission deadlines for CMOs with different risk levels. Note that these deadlines are set based on our empirical study and the analysis of human behaviour in emergent road-traffic situations [55]; however, they can be adjusted based on the VIP safety requirements. For CMOs with a specific risk level, their warning emission deadline is a time point before the estimated collision time, before which the warning message must be emitted. Specifically, we define the warning emission deadline as:

(19) $$t_{dl}=t_{ra}+\delta ,$$

where $t_{ra}$ is an expected reaction time for collision avoidance, and δ is a risk-level specified safety margin factor. According to [55], 70% of road-traffic accidents can be avoided if perceived 1.69∼2.08 s before the collision. For the more vulnerable VIP, we set $t_{ra}$ as 5 s for proper audio message perception and corresponding reaction. To provide additional time for reacting to CMOs, we assign different values of δ (based on our empirical study) to CMOs with different risk levels, i.e., 6 s for ‘severe’, 4 s for ‘serious’, 2 s for ‘minor’, and 0 s for ‘minimal’. In this case, for an identified CMO, its warning message is emitted only when its estimated collision time reaches its warning emission deadline (i.e., $t_{cl}^{\prime }\leq t_{dl}$). Note that if the initial estimated collision time of a CMO is within its warning emission deadline, its warning message is emitted immediately. Complete occlusion by one CMO of another can be a challenge for any vision-based solution. However, this will not affect the overall performance of our scheme because, even with occlusion cases, ARAware can still ensure VIP safety by prioritising and warning the VIP about the visible CMO, which is the closer and more immediate risk in the VIP vicinity. When the VIP avoids the visible CMO, the CMOs behind it will also likely be avoided.

**Early Detection of Small Size CMOs Moving at a Medium-to-High Speed:** It is worth noting that our aim is to detect CMOs as soon as possible to provide early alarms, especially with CMOs moving at a medium-to-high speed, to ensure the safety of the VIP. Therefore, for small size CMOs (i.e., motorbike and bicycle), we consider the detection of the CMO rider since the rider can be detected (as ‘person’) at earlier frames than these CMOs due to their different sizes (i.e., riders are typically taller than their motorbikes or bicycles, thus, more visible). To differentiate between a cyclist and a motorcyclist, as well as a pedestrian, since they all fall under the same class, we set up a classification threshold based on the estimated average moving speed of the CMO $v_{avg}^{\prime }$ (in km/h) on residential roads or a pedestrian walking on sidewalks as follows:

(20) $$CMO\left(v_{avg}^{\prime }\right)=\left\{\begin{array}{cc} motorcyclist, & v_{avg}^{\prime }\geq 20,\\ cyclist, & v_{avg}^{\prime }\geq 10\;\mathrm{and}\;v_{avg}^{\prime }<20,\\ pedestrian, & v_{avg}^{\prime }<10. \end{array}\right.$$

With this method, we manage to detect motorbikes, moving at speeds from 10 km/h to 40 km/h, 1.80 s∼7.20 s earlier, and bicycles, moving at speeds from 8 km/h to 25 km/h, 3.89 s∼12.16 s earlier. It is important to note that this method is only used to increase the distance at which motorbikes and bicycles are detected (discussed later in Section 5.2). Misclassifications here do not affect ARAware performance, as the classification will be corrected in later frames where the CMO is clearly detected. Further, when a warning must be emitted, and misclassification happens, ARAware still can effectively warn about the threat, as the CMO class is not only considered, but the CMO speed and the VIP reaction time are also considered, which are more critical for the safety of VIPs. As part of future work, we plan to retrain YOLOv8 to distinguish between ’cyclists’ and ’motorcyclists’ instead of recognising them as ‘persons’.

**Warning Message Duplication Removal:** Since we extend the detection distance of motorbikes and bicycles, as mentioned above, by detecting their riders, this results in the possibility of producing two bounding boxes (one for the rider and another for the CMO) falling under the same risk level, leading to a duplication of the alerts. To remove such a duplication, WEM uses the Intersection Over Union (IOU) metric to estimate the overlapping between the bounding boxes as follows:

(21) $$IOU_{cmo}=\frac{BB_{i_{1}}^{t}\cap BB_{i_{2}}^{t}}{BB_{i_{1}}^{t}\cup BB_{i_{2}}^{t}},$$

where $BB_{i}^{t}$ represents the bounding box of CMO *i* at time *t*. If $IOU_{cmo}\geq \eta$, WEM triggers one alarm, where η is a predefined threshold indicating an overlap. In our experiments, η=0.25.

**Warning Message Design:** To help VIPs in perceiving potential threats accurately and effectively, for a CMO, its warning message contains the CMO type, risk level, incoming direction, and estimated collision time, which is conveyed to the VIP through the bone conduction headphones using Bluetooth. In particular, we simplify the CMO incoming direction as one of three options using XOtcC in Equation (4), i.e., ‘front’, ‘left’, and ‘right’, for a concise perception. Considering the object’s size, if the CMO is a car, the three directions are identified as follows:

- ‘Front’: XOtcC∈ [−1 m, 1 m].
- ‘Right’: XOtcC >1 m.
- ‘Left’: XOtcC <−1 m.

Otherwise, the three directions are identified as follows:

- ‘Front’: XOtcC ∈ [−0.5 m, 0.5 m].
- ‘Right’: XOtcC > 0.5 m.
- ‘Left’: XOtcC < −0.5 m.

Moreover, the scheme can be adjusted to precisely provide the incoming direction in degrees according to the VIP requirements as follows:

(22) Θ=tan−1XBtcC−XOtcCZBtcC−ZOtcC,

## 4. Implementation and Evaluation

In this section, we present the prototype implementation of ARAware and the methodology used for its evaluation.

### 4.1. ARAware Implementation

**Figure 7:**
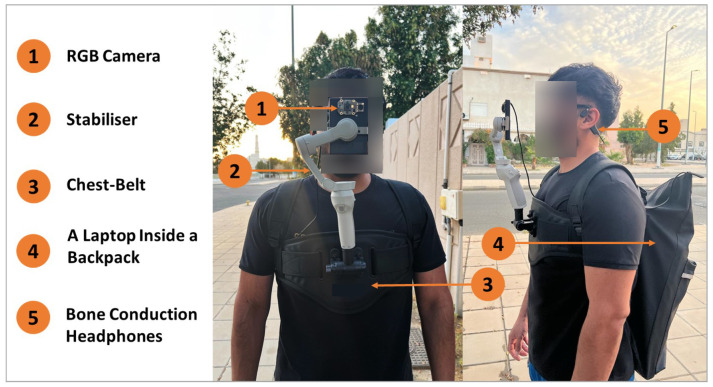
The ARAware real-world prototype.

To evaluate our scheme in a highly practical setting, we construct a real-world prototype (see Figure 7) using commercially available devices. For WVM, we mount an RGB camera, capturing video with a 640 × 480 resolution and a 30 fps frame rate, on a stabilizer to counteract body movements and ensure smooth recording, enabling accurate scene analysis. This stabilizer is securely attached to a comfortable chest belt mount strap to keep the VIP’s hands free. This setup allows the camera to be raised to the VIP’s eye level, providing a close, natural human field of view of the surroundings. The camera (with a size of 50 mm × 28 mm × 0.9 mm and a weight of 5 g) has a fixed but unknown focal length, which is connected to a laptop, in a backpack carried by the VIP, through an USB cable for video transmission. The laptop has an Intel Core i9-9900K CPU, an NVIDIA GeForce RTX 2080 GPU, and 64 GB RAM, where MOT, PM, CRP and WEM are hosted. A laptop is used in our experimentation; however, smaller lighter equipment could be used if we were to be brought to production. Warnings are communicated using bone conduction headphones (chosen, as they do not block ambient sounds, increasing the safety of the VIP) from the WEM using Bluetooth.

We implemented all components of ARAware in Python 3.7.7. Specifically, we used Ultralytics Python library 8.0.145 (https://docs.ultralytics.com/modes/predict/#inference-sources (accessed on 10 May 2024)) to implement the YOLOv8-based object detector. For Deep SORT-based object tracking and DisNet-based distance estimation components, we adapted and employed public implementations (https://github.com/theAIGuysCode/yolov3_deepsort (accessed on 29 April 2024), https://github.com/guanjianyu/DisNet (accessed on 29 April 2024)) based on TensorFlow-gpu 2.1.0 and Keras 2.3.1. Using camera calibration based on OpenCV 4.1 (https://docs.opencv.org/master/dc/dbb/tutorial_py_calibration.html (accessed on 2 April 2024)), we estimated the camera focal length in pixels as $f_{x}=644.83$ px and $f_{y}=644.07$ px, and the camera principal point in pixels as $\left(p_{x},p_{y}\right)$ = (322.97 px, 238.56 px).

### 4.2. Evaluation Methodology

For a comprehensive evaluation, we first conducted extensive real-world experiments (this study is approved by the Engineering and Technology Research Ethics Committee (SETREC) in the Imperial College London) using the ARAware prototype to collect two evaluation datasets (discussed in Section 4.2.1). In these experiments, volunteers (following predetermined scenarios) drove cars, motorbikes, and bicycles and walked at varying speeds while a researcher, mimicking a VIP, captured videos using the prototype. Then, we validated the effectiveness of MOT based on YOLOv8 and Deep SORT, then verified the accuracy of our distance and speed estimation methods, and evaluated the ARAware prototype’s overall performance. Finally, we conducted a comparative analysis between our prototype with the closest state-of-the-art solution, DEEP-SEE [15].

#### 4.2.1. Experimental Dataset

**Figure 8:**
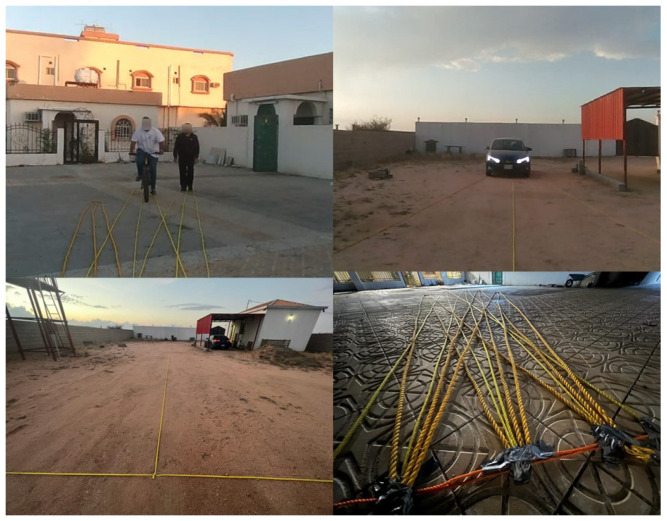
Illustration of video clip collection and experimental environment setting-up.

We recruited up to five volunteers to walk, cycle, and drive in outdoor spaces while a researcher held the ARAware prototype (determined the camera height as 1.52 m), and two datasets were collected:

- **VMOT** contains 96 short video clips with different lengths, totalling 28 min, collected in different private outdoor places (we chose such places to facilitate the setup of our experiments (e.g., as illustrated in Figure 5, identifying the radius of $C_{B}$ and $C_{O}$ and the critical zone on the ground with ropes) for an accurate and comprehensive performance evaluation of our CMO collision prediction method, as well as to ensure the researchers’, volunteers’, and the public’s safety) as shown in Figure 8.
  The video clips encompass different numbers of objects (up to five) from the target classes (car, motorbike, bicycle, pedestrian) located at various distances from the VIP (up to 20 m) and moving at slow speeds (<10 km/h). For all VMOT clips, we manually identified the ground truth (at the frame level) using the MATLAB Ground Truth Labeler (https://uk.mathworks.com/help/driving/ground-truth-labeling.html (accessed on 17 October 2023)). Objects moving inside the critical zone (marked by yellow ropes in Figure 8) were identified as CMOs, and those moving outside the critical zone were identified as non-CMOs. The total numbers of CMOs (i.e., $GT_{c}$) and non-CMOs (i.e., $GT_{Nc}$) are 539 and 217, respectively.
- **VCRP** contains 44 video clips with different lengths, totalling 15 min, collected in private outdoor environments similar to that in VMOT. Each clip specifically focused on one object (e.g., car, motorbike, bicycle, or pedestrian) initially positioned at different distances (up to 100 m) and moving at a uniform fixed speed (up to 60 km/h) towards the camera (i.e., a CMO). Note that our method can be applied to objects moving at varying speeds, and we made the CMO speed uniform during the video only for evaluation purposes, to facilitate the obtaining of accurate speed measurements (i.e., ground truth), as precise speed-measurement equipment was not available. For the object in each clip, both the initial object–VIP distance (i.e., $GT_{D}$) and the object moving speed (i.e., $GT_{S}$) were manually determined, thus known, and the object was tagged with a risk level label according to Figure 6 as the ground truth (i.e., severe, serious, minor, and minimal). Note that in $GT_{S}$ labelling, cars and motorbikes are labelled using their speedometers. For bicycles and pedestrians, we measured the time taken to travel a predefined distance, and then we calculated the speed by dividing the distance by the time taken.

#### 4.2.2. Evaluation Metrics

For performance evaluation, we selected the following evaluation metrics:

- **mean Average Recall** (mAR) indicating the capability of successfully identifying all objects in different classes (in terms of either object types or risk levels):
  (23) $$mAR=\frac{1}{M}\sum_{m=1}^{M}\left(\frac{1}{K}\sum_{k=1}^{K}\frac{TP_{k}^{i}}{TP_{k}^{i}+FN_{k}^{i}}\right),$$
  where *M* is the number of video clips processed, *K* is the number of frames of video clip *m*, and $TP_{k}^{i}$ and $FN_{k}^{i}$ denote numbers of class *i* objects identified and missed in frame *k*, respectively.
- **mean Average Precision** (mAP) indicating the capability of correctly identifying objects in different classes:
  (24) $$mAP=\frac{1}{M}\sum_{m=1}^{M}\left(\frac{1}{K}\sum_{k=1}^{K}\frac{TP_{k}^{i}}{TP_{k}^{i}+FP_{k}^{i}}\right),$$
  where $FP_{k}^{i}$ denotes the number of non-class *i* objects falsely identified as class *i* objects in *k*. We used IOU with a 0.5 threshold to classify the correct and false identifications (i.e., $TP_{k}^{i}$ and $FP_{k}^{i}$).
- **Multiple Object Tracking Accuracy** (MOTA) [56] indicating the capability of correctly tracking objects in their trajectories:
  (25) $$MOTA=\frac{1}{M}\sum_{m=1}^{M}\left(1-\frac{\sum_{k}\left(FP_{k}+FN_{k}+IDSW_{k}\right)}{\sum_{k}GT_{k}}\right),$$
  where $IDSW_{k}$ is the number of object mismatches in a trajectory in *k*, and $GT_{k}$ is the number of ground truth objects in *k*.
- **Multiple Object Tracking Precision** (MOTP) [56] indicating the capability of precisely localising objects in video frames:
  (26) $$MOTP=\frac{1}{M}\sum_{m=1}^{M}\left(\frac{\sum_{j,k}D_{k}^{j}}{\sum_{k}C_{k}}\right),$$
  where $D_{k}^{j}$ denotes how well object *j*’s ground truth and tracked bounding boxes in *k* match with each other, and $C_{k}$ is the number of matched objects in *k*. We used IOU with a 0.5 threshold to classify the matched ground truth and tracked bounding box pairs.
- **Absolute Error** ($E_{A}$) measuring the difference between the estimated and actual values of speed, distance and collision time:
  (27) $$E_{A}=X_{Estimated}-X_{Actual}$$
  Note that the absolute value ($|X_{Estimated}-X_{Actual}|$) is not considered in Equation (27) for more explicit representation, i.e., to show that with a negative value, an estimated value is less than an actual value and vice versa.
- **End-to-End mean FPS** (EmFPS) indicating the capability of achieving real-time CMO identification and classification:
  (28) $$EmFPS=\frac{1}{M}\sum_{m=1}^{M}FPS=\frac{1}{M}\sum_{m=1}^{M}\frac{K_{m}}{t_{m}^{e}-t_{m}^{s}},$$
  where $K_{m}$ denotes clip *m*’s total number of processed frames, and $t_{m}^{e}$ and $t_{m}^{s}$ are the end time and the start time of clip *m*’s end-to-end processing time, respectively.

## 5. Results and Discussions

### 5.1. MOT Validation

To validate the effectiveness of the MOT, we fed the VMOT dataset to the MOT, and collected and analysed the intermediate results of both the YOLOv8-based object detector and the Deep SORT-based object tracker, respectively, which are illustrated in Table 1 and Table 2. Note that such results are collected when MOT detects and tracks all captured objects (i.e., both CMOs and non-CMOs) in VMOT. Considering the performance of YOLOv8 and Deep SORT on other public datasets (e.g., [57,58,59,60]), treated as acceptable in practice, it is obvious that the performance of MOT exceeds this acceptable level on VMOT. Moreover, YOLOv8m (medium variant) outperforms YOLOv8n (nano variant) on VMOT by around 4% mAP and 6% mAR, indicating better object detection. Therefore, we chose YOLOv8m for ARAware’s evaluation to leverage its potentially superior accuracy.

### 5.2. Validation of Distance and Speed Estimation

To validate the performance of our distance and speed estimation methods in Section 3.3 and Section 3.4, respectively, we fed the VCRP dataset to the ARAware prototype, and collected and analysed the intermediate results of distance and speed estimation (i.e., the estimated object–VIP distance ZOtC, and the estimated object speed $v_{avg}^{\prime }$).

#### 5.2.1. Distance Estimation

**Figure 9:**
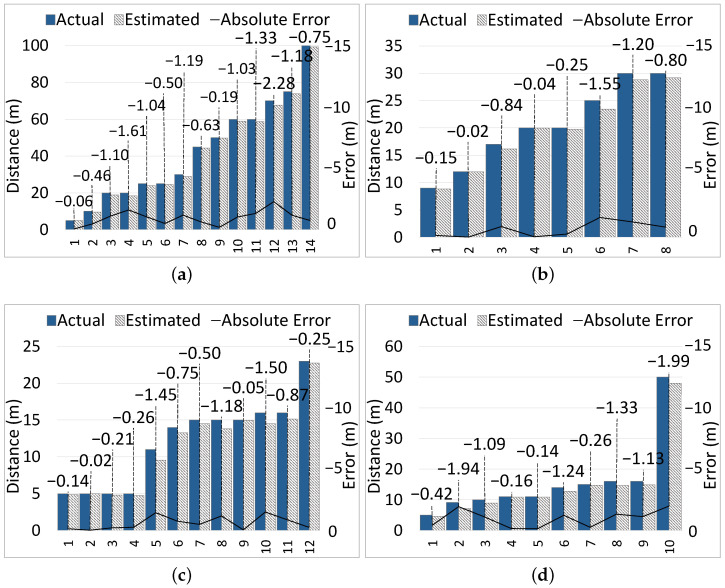
The validation of distance estimation of all four classes in VCRP (44 samples in total). (**a**) Cars, (**b**) motorbikes, (**c**) bicycles, and (**d**) pedestrians.

We collected distance estimation results for each class of CMOs separately. Meanwhile, the initial actual object–VIP distance and the absolute error $E_{A}$ of the estimated distance were collected. We also identified the maximum distance that ARAware was able to detect for each class of CMO. Figure 9a demonstrates the results of 14 samples of the car class at different distances, from 5 m to 100 m. The maximum and average $E_{A}$ of car distance estimation are −2.28 m and −0.95 m, respectively. Note that as mentioned in Section 4.2.2, the negative sign is used only to indicate that the estimated value is less than the actual value. For the motorbike class, Figure 9b shows the results of eight samples at distances from 9 m to 30 m. The maximum and average $E_{A}$ of motorbike distance estimation are −1.55 m and −0.61 m, respectively. For the bicycle class, Figure 9c demonstrates the results of 12 samples at distances from 5 m to 23 m, achieving the maximum $E_{A}$ of −1.50 m and the average $E_{A}$ of −0.60 m. Finally, the results of the pedestrian class are shown in Figure 9d, which contain 10 samples with distances from 5 m to 50 m. The maximum and average $E_{A}$ are −1.99 m and −0.97 m, respectively.

Based on the results, we observe that the accuracy of distance estimation depends on two factors. First is the precision of the predefined values of the average dimensions (i.e., height, width, and depth) of a CMO of a particular class (discussed in Section 3.3). Second is the tightness or alignment of bounding boxes around the detected CMOs. The estimated distance becomes shorter than the actual distance as the size of the object bounding box increases; the error increases. The distance estimation results show that our pre-trained YOLOv8-based detector is reliable even without re-training it with the VCRP dataset. The average $E_{A}$ of distance estimation results over all classes is −0.48 m, which is a negligible error compared to the overall average of the ground truth distances (i.e., 22.67 m). This shows that our pre-defined dimensions of different CMOs classes are accurate enough.

According to VCRP dataset, the maximum distance at which each class of object can be detected is 100 m for cars, 30 m for motorbikes, 23 m for bicycles, and 50 m for pedestrians; see Table 3. Knowing the maximum detectable distance helps us to know the earliest warning time that our scheme can achieve for each class of CMO, and informs of the warning notification deadlines. In fact, in our experiments, different classes of objects have different maximum detectable distances, majorly due to their different sizes. For example, cars are the widest among all classes of objects, which enables them to be detectable at distances up to 100 m. Although the width of pedestrians is only 45∼55 cm in average, they can be detected up to 50 m away since they are high (170∼175 cm in average). Motorbikes and bicycles are detected at shorter distances due to their smaller widths than cars and shorter heights than pedestrians.

According to [55], the reaction time for pedestrians to avoid 70% of perceived road-traffic collisions is 1.69∼2.08 s. Therefore, considering the average speed limit of cars in urban roads (i.e., 38∼45 km/h [54]), we believe that the 100 m detectable distance of cars is sufficient, where ARAware is able to emit an alarm 8∼9.47 s earlier than the potential collision. For motorbike and bicycles, the detection of their riders (as persons, mentioned in Section 3.5) at 50 m contributes to extending the time of alarm emission, where ARAware can warn 4∼4.73 s and 8.99∼11.99 s before the potential collision, considering the 38∼45 km/h and 15∼20 km/h average speeds, respectively. It is obvious that ARAware manages to leave significantly more time for the VIP to react and avoid predicted collisions compared to [55].

#### 5.2.2. Speed Estimation

We collected speed estimation results for all classes of CMO when the camera (as well as the VIP) was either static or moving. Specifically, we collected the actual and estimated average speeds, and the absolute error between them, for each class of CMOs separately.

**Figure 10:**
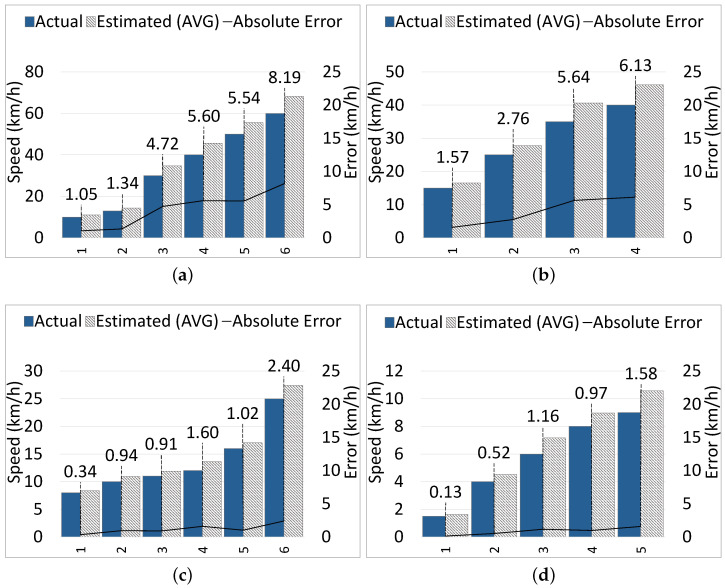
The validation of speed estimation of all four classes in VCRP with static camera (21 out of 44 samples, in total). (**a**) Cars, (**b**) motorbikes, (**c**) bicycles, and (**d**) pedestrians.

**Static Camera:** Figure 10a shows the results of six samples from the car class, which are moving at speeds from 10 km/h to 60 km/h. The maximum $E_{A}$ is 8.19 km/h when the car was moving at 60 km/h; the average $E_{A}$ across all car samples is 4.41 km/h. Figure 10b shows the results of four samples from the motorbike class, which were moving at speeds from 15 km/h to 40 km/h. The maximum $E_{A}$ is 6.13 km/h when the motorbike was moving at 40 km/h, and the average $E_{A}$ is 4.03 km/h. In Figure 10c, the results of six bicycle samples moving at speeds from 8 km/h to 25 km/h are illustrated. The $E_{A}$ ranges from 0.34 km/h to 2.40 km/h, with an average of 1.20 km/h. Finally, five pedestrian samples moving at speeds from 1.5 km/h to 9 km/h are demonstrated in Figure 10d, and the $E_{A}$ ranges from 0.13 km/h to 1.58 km/h, achieving an average of 0.87 km/h.

**Figure 11:**
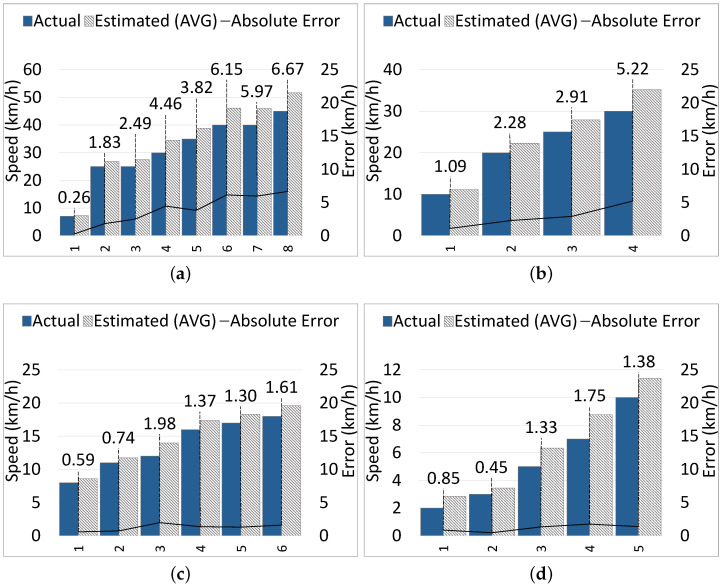
The validation of speed estimation of all four classes in VCRP with moving camera (23 out of 44 samples, in total). (**a**) Cars, (**b**) motorbikes, (**c**) bicycles, and (**d**) pedestrians.

**Moving Camera:** In our experiments, the VIP (and the camera) was moving at a reasonable speed (≤3 km/h). Figure 11a shows the results of eight car samples moving at speeds from 7 km/h to 45 km/h. The maximum $E_{A}$ is 6.67 km/h when the car was moving at 45 km/h; the average $E_{A}$ is 3.96 km/h. Figure 11b presents the results of four motorbike samples moving at speeds from 10 km/h to 30 km/h. The maximum $E_{A}$ is 5.22 km/h when the motorbike was moving at 30 km/h, and the average $E_{A}$ is 2.88 km/h. In Figure 11c, six bicycle samples are presented, which travel at speeds from 8 km/h to 18 km/h, and the error ranges from 0.59 km/h to 1.98 km/h, with an average of 1.27 km/h. For the pedestrian class, the results of five samples moving at speeds from 2 km/h to 10 km/h are illustrated in Figure 11d, and the error ranges from 0.45 km/h to 1.75 km/h, with an average of 1.15 km/h.

According to the results, we observe that the speed estimation error increases when the CMO moving speed increases. With both static and moving cameras, for high-speed objects like cars and motorbikes, the speed estimation error is relatively higher compared to low-speed objects, bicycles and pedestrians. For bicycles and pedestrians, the error is negligible, and there is no obvious difference between error rates of different classes of CMOs (e.g., bicycles and pedestrians) moving at low-to-medium speeds (e.g., <25 km/h).

Generally, there are three requirements to achieve accurate speed estimation with ARAware, i.e., the presence of distinctive and good enough features in the surrounding scenes, the stable mounting of the camera preventing unstable movements or strong shaking, and the reasonably slow moving speed of the VIP (≤3 km/h). We require the VIP to move at a slow speed, with a stable mounted camera, only to obtain accurate results, as our speed estimation method depends on extracting features from the background, which is affected by camera movement. We consider this as acceptable, as it matches the research on VIP walking patterns, which indicates that VIPs generally walk at speeds of <5 km/h, with a further decrease in speed observed in complex or unfamiliar environments [47]; therefore, it will not hinder them. Across all classes (for both static and moving camera cases), the average speed estimation absolute error is 2.47 km/h, which is a relatively low error (12.90%) compared to the overall average of the ground truth speeds, which is 19.14 km/h. Considering that, for all experiments, the absolute error of speed estimation is positive, which means that the estimated speed is always slightly higher than the actual moving speed of CMOs, we accept this low error, as it contributes to obtaining estimated collision times that are shorter than actual collision times, and thus emitting earlier notifications and leaving more time for VIPs to react.

### 5.3. Performance of ARAware

We validated the effectiveness of our design, and we evaluated the ARAware performance with real-time CMO identification and classification. Specifically, we fed the VMOT and the VCRP to ARAware for end-to-end (E2E) CMO identification and classification, respectively. For overall efficiency, ARAware achieves a 32 EmFPS and manages to process the incoming 30 fps video stream in real-time, with an average E2E processing time of 0.031 s. This falls well within the real-time processing threshold of ≤0.033 s (33 ms) outlined in Section 1.

#### 5.3.1. CMO Identification

**Figure 12:**
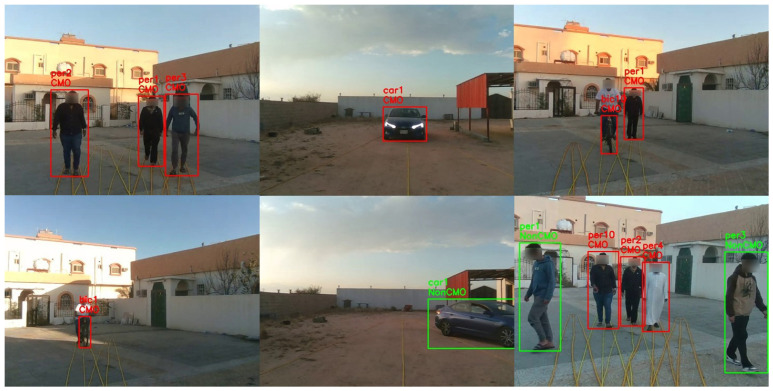
Illustration of CMO identification on VMOT.

The overall and detailed results of CMO identification are illustrated in Table 4 and Table 5, respectively. As we can see, when identifying all types of CMOs, ARAware manages to achieve an overall mAP of 88.20% and an overall mAR of 97.26% over all CMO classes. For the mAP, cars achieve the highest mAP, followed by motorbikes, bicycles, and pedestrians. Pedestrians have the lowest mAP due to their slower movement than other CMOs. Since the time difference between captured frames is negligible (0.033 s), the positions of slow-moving objects like pedestrians experience minimal change or remain the same. This affects the precision of determining the objects’ moving direction (i.e., angle β of vector $\vec{OO^{\prime }}$ in Figure 5), which leads to more false positive cases (FP) and, thus, lower mAP. Additionally, for different classes of CMOs, when their general moving speed decreases (i.e., from cars to pedestrians), the mAR increases, as the missed cases (false negative, FN) decrease. This means the CMO speed mainly influences the mAR. However, knowing that CMO identification is evaluated at every frame, missed cases are limited to a few frames (for negligible time), which is basically caused by the MOT module (i.e., Deep SORT tracker). This very short time window where objects are missing will not affect our work, as ARAware successfully identified all CMOs in all VMOT video clips without completely missing a CMO in a whole clip. Figure 12 illustrates the successful CMO identification by ARAware in real-world scenarios with varying numbers of CMOs.

#### 5.3.2. CMO Classification

**Figure 13:**
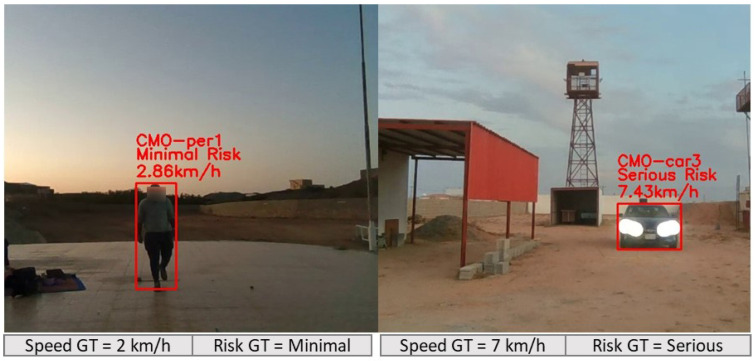
Illustration of CMO classification On VCRP.

The overall and detailed results of CMO classification are illustrated in Table 4 and Table 6, respectively. When classifying CMOs into different risk levels illustrated in Figure 6, ARAware manages to achieve an 91.69% overall mAP and an 100% overall mAR. This demonstrates the effectiveness of ARAware in CMO classification, especially for high-risk objects, like cars (99.32% mAP) and motorbikes (96.01% mAP). For pedestrians and bicycles, although their mAP is lower than cars and motorbikes, it is still more than acceptable, as they are low-risk objects. Figure 13 shows ARAware’s ability to estimate the risk level of CMOs in real-world scenarios.

#### 5.3.3. Warning Notification

In Table 7, we show the performance of ARAware’s warning emission for one video that includes four CMOs with different collision times and risk levels. The table shows the CMOs with their actual risk levels $R_{l}$, actual collision time $t_{cl}$, estimated collision time $t_{cl}^{\prime }$, warning priorities assigned by ARAware $Pri._{w}$, the order in which the warning messages were sent $Ord._{w}$, and when its warning message was actually sent by ARAware $Emi._{w}$. It is clear that $t_{cl}^{\prime }$ of all classes is less than $t_{cl}$ with an average $E_{A}$ of −2.83 s. This means that ARAware manages to protect the safety of the VIP by emitting earlier warnings, since the CMOs estimated collision time is earlier than the actual collision time by almost 3 s on average. Further, ARAware manages to prioritise the warning communication based on the CMO risk level and collision time. It immediately emits warnings for higher-risk CMOs (i.e., cars and motorbikes), and reduces false alarms by setting deadlines for lower risk CMOs (e.g., for pedestrians, a warning emission deadline of 5 s is set, and the warning message is postponed for 15.21−5=10.21 s).

Table 8 demonstrates the setup of warning emission deadlines (see Section 3.5) based on the maximum detectable distance of each CMO and the actual moving speed considered in our experiments (see Section 5.2). The table shows CMOs with their risk levels $R_{l}$, the earliest time that they can be identified before the predicted collision $Earl._{dt}$, the expected reaction time $t_{ra}$, the risk-level specified safety margin factor δ, the warning emission deadline $t_{dl}$, and when warning messages should be sent by ARAware $Emi._{w}$. It is obvious that for severe risk levels, $t_{dl}$ is achievable for cars moving at speeds from 25 km/h to 34 km/h, while it is not achievable for motorbikes moving at speeds from 25 km/h to 40 km/h, as their detectable distance is less than that of cars by 50 m as discussed in Section 5.2. However, ARAware manages to address this issue by always emitting warning emissions immediately for motorbikes to guarantee the safety of the VIP. We believe that with ARAware, there is more than an acceptable amount of time (generally 1.69∼2.08 s according to [55]) for the VIP to react and void collisions. For other risk levels, $t_{dl}$ is achievable for all classes of CMOs. This means that ARAware’s warning emission strategy manages to efficiently handle CMOs with such different risk levels.

Audible warning messages are transmitted to the bone conduction headphones of the VIP via Bluetooth, informing the VIP about the CMO type, risk level, incoming direction, and estimated collision time, for clear perception of the upcoming threat.

### 5.4. ARAware versus DEEP-SEE

This section compares our proposed scheme with the state-of-the-art DEEP-SEE, focusing on key common components like object tracking, collision identification, and object warning and risk classification; the overall efficiency of both schemes is evaluated as well. Like ARAware, DEEP-SEE utilises computer vision to aid VIP mobility outdoors. While using different approaches, both schemes identify collisions with various obstacles (cars, motorbikes, bicycles, and pedestrians), classify their risk, and prioritise warnings based on their risk level. However, unlike ARAware, DEEP-SEE lacks capabilities in per-object distance estimation (it only estimates the distance to the first visible area on the floor), speed estimation, and collision time prediction.

While open-source code for DEEP-SEE was not available, we carefully followed the authors’ methodologies described in their original work [15] to re-implement DEEP-SEE using Python 3.7.7. However, certain DEEP-SEE components, such as GOTURN [62] and DeepCompare [63], were themselves built based on other prior works. Therefore, instead of rebuilding and retraining them from scratch, we leveraged existing open-source PyTorch implementations for these specific components (https://github.com/amoudgl/pygoturn/tree/master (accessed on 6 November 2023), https://github.com/szagoruyko/cvpr15deepcompare (accessed on 10 November 2023)). This is to speed up the implementation process while ensuring the reliability of obtained results by relying on well-established components.

Generally, both the ARAware and DEEP-SEE trackers are constructed based on the well-known YOLO object detector. For a fair comparison, YOLOv8, the same version used with ARAware’s Deep SORT-based tracker, is also used to feed input data to DEEP-SEE’s adapted GOTURN-based tracker, and the VMOT dataset feeds both trackers. Table 9 compares the performance of both trackers in terms of MOTA, MOTP, and the average processing time. The comparison demonstrates that ARAware outperforms DEEP-SEE in accurately and precisely tracking multiple objects, where it achieves both higher 94.55% MOTA and 80.88% MOTP. Note, as the DEEP-SEE tracker does not directly assign IDs to tracked objects, unlike the ARAware tracker, we conservatively assumed zero ID switches (i.e., assuming no object identity changes with DEEP-SEE) for MOTA comparison. However, even under this potentially optimistic assumption for DEEP-SEE, ARAware achieved a superior MOTA score, emphasising its robust performance in objects tracking. Additionally, ARAware exhibits significantly faster tracking (0.64 ms on average) than DEEP-SEE (380 ms, exceeding the real-time threshold of ≤33 ms, see Section 1), both running on an NVIDIA GeForce RTX 2080 GPU. This notable difference is attributed to the computationally intensive tracking of DEEP-SEE, making it less suitable for time-sensitive scenarios, unlike ARAware.

Different from ARAware, DEEP-SEE lacks individual distance estimates for objects. Instead, it estimates the distance to the first visible area on the floor and defines a ‘trapezium of interest’ based on the camera’s height and angle. This trapezium acts as a safety zone to identify potential collisions and obstacle proximity to the VIP in the real world. Based on our camera’s parameters (height of 1.52 m and angle of 74.9°), the estimated distance for the safety zone is approximately 5 m. For potential collision identification, DEEP-SEE classifies an object inside the safety zone as ‘urgent’; otherwise, it is classified as ‘normal’. To effectively evaluate both schemes in collision identification, we treated ‘urgent’ objects as ’CMO’ and ’normal’ objects as ‘non-CMO’; this is to align with our classification for collision prediction (see Section 3.4.1). Table 10 compares the performance of CMO identification between ARAware and DEEP-SEE (on the VMOT dataset). ARAware achieves higher mAP (by 10.88%) than DEEP-SEE. This indicates that ARAware more accurately identifies potential collisions across different scenarios and object types. Additionally, ARAware achieves an impressive mAR of 97.26%, significantly higher than the DEEP-SEE mAR of 54.64%. This means ARAware misses far fewer true CMOs, ensuring better recall and more reliable collision detection. This is because ARAware goes beyond merely the relative position of objects to the VIP, unlike DEEP-SEE, i.e., it analyses the crucial factor, which is the objects’ moving angle. This grants ARAware a deeper understanding of objects’ behaviour, predicting potential collision threats effectively, and all this with an impressive average E2E processing time of 31 ms, 93% faster than DEEP-SEE, at 430 ms, both running on the same machine. Additionally, the DEEP-SEE safety zone of 5 m is inadequate for identifying potential collisions and ensuring VIP safety, particularly with objects moving at high speeds.

Despite the limited VIP safety zone, DEEP-SEE prioritises warning the VIP about ’normal’ cars and motorbikes (outside the safety zone) over low-risk ’urgent’ objects (i.e., pedestrians) to enhance VIP safety. However, DEEP-SEE still cannot effectively prevent collisions. This is because, unlike ARAware, DEEP-SEE cannot estimate object speed, which hampers its adaptation to changes in objects’ velocities, potentially compromising VIPs safety. Object speed is crucial factor to timely warn the VIP about potential threats. To illustrate this, consider one car and one bicycle: the car is moving at 45 km/h and is located 20 m away, while the bicycle is moving at 3 km/h and 5 m away. With DEEP-SEE, the bicycle is classified as ’urgent’, as it is inside the safety zone of the VIP, whereas the car is labelled as ‘normal’. Based on the DEEP-SEE warning prioritisation strategy, the VIP is alerted only to the most critical threat, the bicycle. However, this poses a significant danger to the VIP, as the bicycle takes 6 s to approach, while the car takes only 1.6 s before the collision happens. In contrast, ARAware promptly warns the VIP about the car since it is classified as *’severe’* CMO as its speed is ≥25 km/h and its estimated collision time is less than the bicycle (classified as *’minor’* CMO as its speed is <10 km/h). This shows that ARAware’s risk classification and prioritisation of warning message are more effective than DEEP-SEE, providing a safer solution for VIPs.

Moreover, with DEEP-SEE, frequent false alarms can be issued, which could desensitize the VIP to real threats. To show this, we recorded a video for a car approaching the VIP at 5 km/h, initially 30 m away, with a potential collision in 21.58 s. At the 12 m distance (potential collision in 8.63 s), the car safely moves away from the VIP’s path. According to the DEEP-SEE warning prioritisation, the car is alerted at first detection (30 m), even if it is outside the 5 m safety zone. In this case, DEEP-SEE generates false alarms for over 12.95 s (21.58 s − 8.63 s = 12.95 s) until the car leaves the recorded video frame as the car’s moving direction is ignored. This can distract the VIPs from genuine threats that might require immediate action. In contrast, with ARAware, the car is alerted 9 s ahead (when deadline is satisfied, see Section 3.5), instead of 21.58 s in advance when it is first identified, and the alert lasts for only around 0.4 s, and it is promptly stopped once the car changes the moving direction and classified as ‘non-CMO’. This demonstrates the effectiveness of ARAware in reducing false alarms while emitting timely alarms, unlike DEEP-SEE.

To summarise, based on the findings presented above, ARAware surpasses the state-of-the-art approach DEEP-SEE [15] by accurately tracking objects and effectively identifying potential collisions. Additionally, different from DEEP-SEE, ARAware achieves finer-grained object risk level estimation, and thus more effective warning message prioritisation. By setting up risk-specific warning deadlines, ARAware also achieves a better trade-off between timely warning and false alarm reduction. All this is accomplished with an impressive 93% faster E2E processing speed than DEEP-SEE.

## 6. Limitation of ARAware

The approaches provided in this work substantively improve the performance of assistance technologies for VIP; however, ARAware can be further optimised in terms of motion detection, the prototype’s form factors and user interface, and scalability.

### 6.1. Motion Detection

Our motion detection is a combination of trajectory classification-based and object tracking-based methods [64], i.e., it relies on the DEEP-SORT tracker to localize objects and the ORB extractor to extract features from a scene background to distinguish between movements caused by moving objects and those caused by the camera, see Section 3.2.3. Compared to background modelling-based methods [64], our method is computationally less complex, as it does not require background modelling and homography transformations; this helps to achieve faster performance. As discussed at the end of Section 4, while our scheme compensates for camera movement, the effectiveness of our motion detector can deteriorate if the camera is moved faster than normal VIP walking speeds (e.g., abrupt movements and strong camera shaking), or if there are a lack of distinctive features in a scene. However, there exists work that deals with such movements [65] and better discriminates features in challenging scenes [66] that could be investigated to improve our motion detector; this is future work.

### 6.2. Prototype Endurance and Human-Machine Interface

Our real-world prototype used a high-performance laptop placed in a backpack in our experiments, and this was not without limitations. For example, the scheme we present has room to make use of more energy-efficient algorithms given the mobile nature of the solution. Therefore, for future work, approaches such as light-weight neural networks [67] for mobile devices and light version of YOLOv8 (e.g., YOLOv8n) [37] could be investigated, and the use of deep learning specific hardware like the OpenNCC NCB (https://www.crowdsupply.com/eyecloud/openncc-ncb (accessed on 27 June 2024)) have the potential to significantly improve the usability of ARAware.

Further, the ARAware scheme provides an effective warning strategy to emit timely alarms and convey descriptive acoustic warning messages to the VIP regarding surrounding threats. In the ARAware prototype, we chose bone conduction headphones to not block out ambient sounds, as VIPs tend to make more use of sound to generally navigate. However, this first prototype of ARAware inherently needs 1∼2 s to generate and deliver warning messages, mainly due to the text-to-speech conversion of warning messages. Future work will specifically focus on a rapid and effective alert mechanism, proportional to the severity of surrounding threats, while guaranteeing concise threat perceptions will significantly improve the performance of ARAware’s prototype warning emission. Perhaps encoding the instruction into a signal or more focus on what makes sense to the VIP requires further HCI-focused study. To this end, we are investigating and evaluating different VIP warning methods [8,68] for a better trade-off between fast alarms and effective VIP perception.

### 6.3. Scalability

While experimental results show that ARAware effectively works with real-world outdoor scenarios involving multiple objects (up to 5) from different classes, including cars, motorcycles, bicycles, and pedestrians, future work will conduct more extensive experiments to investigate its performance with more complex scenarios (e.g., crowded environments) and under different light conditions. It also will explore edge computing and distributed processing [69] for handling increased object density and maintaining real-time performance.

## 7. Conclusions

In this paper, we present the ARAware scheme to practically assist the mobility of a visually impaired person in challenging outdoor environments through real-time CMO identification and fine-grained CMO risk estimation, as well as timely and usable CMO warning. In constructing the E2E CMO avoidance pipeline, we explicitly explored the design and optimisation of all scheme modules considering the VIP properties and perceptions in practice. In particular, we designed a deep learning-based CMO identifier capable of analysing incoming real-time video without losing critical information. We constructed a 2D to 3D coordinate projection method to enhance the accuracy of our collision and risk predictor. Through extensive empirical study, we proposed a CMO risk estimation and classification method, and a risk-aware prioritised CMO warning strategy that achieves a subtle trade-off between VIP safety and scheme usability. Extensive prototype-based experiments in different real-world scenarios demonstrate the effectiveness of ARAware in terms of real-time and accurate CMO identification (achieving 97.26% overall mAR and 88.20% overall mAP with 30 fps input video and 32 fps processing speed) and precise CMO risk level classification (achieving 100% overall mAR and 91.69% overall mAP), and illustrate the trade-offs between in-time warnings and false alarm reduction. Finally, we believe ARAware is the first step towards developing a fully practical assistance system for VIPs, and through work in HCI and further algorithmic and hardware assist improvements can easily inform the design of the ARAware prototype in a form factor that better suits the mobility requirements of real people.

## Figures and Tables

**Table 1:** Performance of YOLOv8-based object detector on VMOT with two different sizes.

Model	Dataset	mAP_50_	mAR
YOLOv8m	VMOT	97.61%	96.45%
YOLOv8m [37]	COCO [57]	50.20%	-
YOLOv8n	VMOT	93.68%	90.15%
YOLOv8n [37]	COCO [57]	37.30%	-
YOLOv8n [38]	CrowdHuman [58]	82.00%	-

**Table 2:** Performance of Deep SORT-based object tracker on VMOT.

Model	Dataset	MOTA	MOTP
Deep SORT (YOLOv8m)	VMOT	94.55%	80.88%
Deep SORT (YOLOV5l) [61]	UA-DETRAC [59]	84.40%	77.40%
Deep SORT (Faster R-CNN) [39]	MOT16 [60]	61.40%	79.10%

**Table 3:** Performance of distance and speed estimation on VCRP ($C_{st}$: camera status, $GT_{S}$: actual speed, $Est._{S}$: estimated speed, $SD_{S}$: standard deviation, $GT_{D}$: actual distance, $E_{A}$: absolute error, and $E_{R}$: relative error).

Samp.	CMO	$C_{\mathrm{st}}$	${\mathrm{GT}}_{S}$ (km/h)	$\mathrm{Est}._{S}$ (km/h)	${\mathrm{SD}}_{S}$ (km/h)	${\mathrm{GT}}_{D}$ (m)	$E_{A}$ (m)	$E_{R}$ (m)
1	Car	M	7	7.26	0.13	5	−0.06	−0.01
2		S	10	11.05	3.13	10	−0.46	−0.05
3		S	13	14.34	4.81	20	−1.10	−0.06
4		M	25	26.83	2.09	20	−1.61	−0.08
5		M	25	27.49	3.62	25	−1.04	−0.04
6		S	30	34.72	3.56	25	−0.50	−0.02
7		M	30	34.46	3.31	30	−1.19	−0.04
8		M	35	38.82	1.44	45	−0.63	−0.01
9		S	40	45.60	3.03	50	−0.19	−0.00
10		M	40	46.15	3.33	60	−1.03	−0.02
11		M	40	45.97	2.40	60	−1.33	−0.02
12		M	45	51.67	2.74	70	−2.28	−0.03
13		S	50	55.54	6.38	75	−1.18	−0.02
14		S	60	68.19	2.86	100	−0.75	−0.01
15	Motorbike	M	10	11.09	0.32	9	−0.15	−0.02
16		S	15	16.57	6.49	12	−0.02	−0.00
17		M	20	22.28	2.11	17	−0.84	−0.05
18		S	25	27.76	5.63	20	−0.04	−0.00
19		M	25	27.91	3.57	20	−0.25	−0.01
20		M	30	35.22	2.48	25	−1.55	−0.06
21		S	35	40.64	3.57	30	−1.20	−0.04
22		S	40	46.13	4.59	30	−0.80	−0.03
23	Bicycle	S	8	8.34	3.50	5	−0.14	−0.03
24		M	8	8.59	0.82	5	−0.02	−0.00
25		S	10	10.94	1.66	5	−0.21	−0.04
26		S	11	11.91	2.34	5	−0.26	−0.05
27		M	11	11.74	1.74	11	−1.45	−0.13
28		M	12	13.98	0.65	14	−0.75	−0.05
29		S	12	13.60	0.85	15	−0.50	−0.03
30		S	16	17.02	4.09	15	−1.18	−0.08
31		M	16	17.37	2.04	15	−0.05	−0.00
32		M	17	18.30	4.22	16	−1.50	−0.09
33		M	18	19.61	2.61	16	−0.87	−0.05
34		S	25	27.40	3.59	23	−0.25	−0.01
35	Pedestrian	S	1.5	1.63	0.08	5	−0.42	−0.08
36		M	2	2.85	0.15	9.15	−1.94	−0.21
37		M	3	3.45	0.19	10	−1.09	−0.11
38		S	4	4.52	0.97	11	−0.16	−0.01
39		M	5	6.33	0.95	11	−0.14	−0.01
40		S	6	7.16	1.23	14	−1.24	−0.09
41		M	7	8.75	0.48	15	−0.26	−0.02
42		S	8	8.97	1.97	16	−1.33	−0.08
43		S	9	10.58	0.19	16	−1.13	−0.07
44		M	10	11.38	0.81	50	−1.99	−0.04

**Table 4:** Overall performance of ARAware.

Component	Over. mAP	Over. mAR	EmFPS
CMO identification	88.20%	97.26%	32 fps
CMO classification	91.69%	100%	

**Table 5:** Performance of ARAware-based CMO identification on VMOT.

CMO	${\mathrm{GT}}_{c}$	${\mathrm{GT}}_{\mathrm{Nc}}$	TP	FP	FN	TN	mAP	mAR
Car	182	65	174	19	8	46	91.61%	95.62%
Motorbike	112	31	108	19	4	12	88.96%	96.62%
Bicycle	77	35	75	17	2	18	86.44%	96.97%
Pedestrian	168	86	167	46	1	40	85.81%	99.83%
Total	539	217	524	101	15	116	88.20%	97.26%

**Table 6:** Performance of ARAware-based CMO classification on VCRP.

CMO	TP	FP	mAP	Over. mAP
Car	422	4	99.32%	91.69%
Motorbike	105	7	96.01%	
Bicycle	159	47	88.77%	
Pedestrian	737	194	82.66%	

**Table 7:** Performance of ARAware’s warning emission.

CMO	$R_{l}$	$t_{\mathrm{cl}}$ (s)	$t_{\mathrm{cl}}^{\prime }$ (s)	$\mathrm{Pri}._{w}$	$\mathrm{Ord}._{w}$	$\mathrm{Emi}._{w}$
Car	Serious	10	7.38	2	2	Imm.
Moto._*R*_	Serious	5.40	2.70	2	1	Imm.
Bic._*R*_	Minor	9.95	7.60	3	3	$t_{cl}^{\prime }\leq 7$ s
Ped.	Minimal	18.86	15.21	4	4	$t_{cl}^{\prime }\leq 5$ s

**Table 8:** Warning emission deadline setups.

CMO	$R_{l}$	$\mathrm{Earl}._{\mathrm{dt}}$ (s)	$t_{\mathrm{ra}}$ (s)	δ (s)	$t_{\mathrm{dl}}$ (s)	$\mathrm{Emi}._{w}$
Car	Severe	6–14.41	5	6	11	Imm. or $t_{cl}^{\prime }\leq t_{dl}$
	Serious	14.99–51.55	5	4	9	$t_{cl}^{\prime }\leq t_{dl}$
Moto._*R*_	Severe	4.50–7.20	5	6	11	Imm.
	Serious	7.50–17.99	5	4	9	Imm. or $t_{cl}^{\prime }\leq t_{dl}$
Bic._*R*_	Serious	7.20–17.99	5	4	9	Imm. or $t_{cl}^{\prime }\leq t_{dl}$
	Minor	20–22.52	5	2	7	$t_{cl}^{\prime }\leq t_{dl}$
Ped.	Minor	17.99–25.77	5	2	7	$t_{cl}^{\prime }\leq t_{dl}$
	Minimal	29.94–119.05	5	0	5	$t_{cl}^{\prime }\leq t_{dl}$

**Table 9:** Object tracking performance evaluation: ARAware’s Deep SORT-based tracker vs. DEEP-SEE’s GOTURN-based tracker.

Model	MOTA	MOTP	Aver. Processing Time
Deep SORT (YOLOv8)	94.55%	80.88%	0.64 ms
DEEP-SEE [15] (YOLOv8)	87.04%	68.75%	380 ms

**Table 10:** CMO identification performance evaluation: ARAware vs. DEEP-SEE.

Scheme	Over. mAP	Over. mAR	Aver. E2E Processing Time
ARAware	88.20%	97.26%	31 ms
DEEP-SEE [15]	77.32%	54.64%	430 ms

## Data Availability

The data presented in this study are available on request from the corresponding author, H.S., due to privacy and ethical reasons.

## Abbreviations

The following abbreviations are used in this manuscript:

VIPs	Visually Impaired People
CMO	Critical Moving Object
WVM	Wearable Vision Module
MOT	Moving Object Tracker
PM	2D-to-3D Projection Module
CRP	Collision and Risk Predictor
WEM	Warning Emission Module
CCS	Camera Coordinate System
PCS	Pixel Coordinate System

## Appendix A. Derivation of the Alpha Angle

We calculate the angle ∠α between $\bar{AB}$ and $\bar{AB_{1}}$ as shown in Figure 5. Obviously, since $▵ABB_{1}$ and $▵AOO_{1}$ are similar, there is:

(A1) $$\frac{|\bar{AB}|}{|\bar{AO}|}=\frac{r_{B}}{r_{O}},$$

(A2) $$\frac{|\bar{AB}|}{|\bar{AO}|}=\frac{|\bar{\left(AO+OB\right)}|}{|\bar{AO}|}=\frac{|\bar{AO}|+d}{|\bar{AO}|}=\frac{r_{B}}{r_{O}},$$

thus,

(A3) $$|\bar{AO}|\;=\frac{r_{O}}{r_{B}-r_{O}}d,$$

From the Pythagoras equation, there is:

(A4) $$|\bar{AO_{1}}|\;=\sqrt{|\bar{AO}{|}^{2}-{r_{O}}^{2}}.$$

Inserting Equation (A3) into Equation (A4), there is:

(A5) $$|\bar{AO_{1}}|\;=r_{O}\sqrt{\frac{d^{2}}{{\left(r_{B}-r_{O}\right)}^{2}}-1}.$$

For α in $▵AOO_{1}$, there is:

(A6) $$tan\left(\alpha \right)=\frac{r_{O}}{|\bar{AO_{1}}|}=\frac{1}{\sqrt{\frac{d^{2}}{{\left(r_{B}-r_{O}\right)}^{2}}-1}},$$

thus,

(A7) $$\alpha =tan^{-1}\left(\frac{1}{\sqrt{\frac{d^{2}}{{\left(r_{B}-r_{O}\right)}^{2}}-1}}\right).$$

## References

[1] El-Taher F.E.Z., Miralles-Pechuán L., Courtney J., Millar K., Smith C., Mckeever S. (2023). A survey on outdoor navigation applications for people with visual impairments. IEEE Access.

[2] Hafeez F., Sheikh U.U., Al-Shammari S., Hamid M., Khakwani A.B.K., Arfeen Z.A. (2023). Comparative analysis of influencing factors on pedestrian road accidents. Bull. Electr. Eng. Inform..

[3] Islam M. (2023). An exploratory analysis of the effects of speed limits on pedestrian injury severities in vehicle-pedestrian crashes. J. Transp. Health.

[4] University of Zurich (2024). Bio-Inspired Cameras and AI Help Drivers Detect Pedestrians and Obstacles Faster. https://www.sciencedaily.com/releases/2024/05/240529144230.htm.

[5] Akamine S., Totoki S., Itami T., Yoneyama J. (2022). Real-time obstacle detection in a darkroom using a monocular camera and a line laser. Artif. Life Robot..

[6] Mala N.S., Thushara S.S., Subbiah S. Navigation gadget for visually impaired based on IoT. Proceedings of the 2017 2nd International Conference on Computing and Communications Technologies (ICCCT’17).

[7] Beingolea J.R., Zea-Vargas M.A., Huallpa R., Vilca X., Bolivar R., Rendulich J. (2021). Assistive devices: Technology development for the visually impaired. Designs.

[8] Kayukawa S., Higuchi K., Guerreiro J., Morishima S., Sato Y., Kitani K., Asakawa C. Bbeep: A sonic collision avoidance system for blind travellers and nearby pedestrians. Proceedings of the 2019 CHI Conference on Human Factors in Computing Systems.

[9] El-Taher F.E.Z., Taha A., Courtney J., Mckeever S. (2021). A Systematic Review of Urban Navigation Systems for Visually Impaired People. Sensors.

[10] Schieber H., Kleinbeck C., Pradel C., Theelke L., Roth D. (2022). A mixed reality guidance system for blind and visually impaired people. Proceedings of the 2022 IEEE Conference on Virtual Reality and 3D User Interfaces Abstracts and Workshops (VRW).

[11] Muhsin Z.J., Qahwaji R., Ghanchi F., Al-Taee M. (2024). Review of substitutive assistive tools and technologies for people with visual impairments: Recent advancements and prospects. J. Multimodal User Interfaces.

[12] Rodrigo-Salazar L., González-Carrasco I., Garcia-Ramirez A.R. (2021). An IoT-based contribution to improve mobility of the visually impaired in Smart Cities. Computing.

[13] Asiedu Asante B.K., Imamura H. (2023). Towards Robust Obstacle Avoidance for the Visually Impaired Person Using Stereo Cameras. Technologies.

[14] Lin B., Lee C., Chiang P. (2017). Simple smartphone-based guiding system for visually impaired people. Sensors.

[15] Tapu R., Mocanu B., Zaharia T. (2017). DEEP-SEE: Joint object detection, tracking and recognition with application to visually impaired navigational assistance. Sensors.

[16] Ou W., Zhang J., Peng K., Yang K., Jaworek G., Müller K., Stiefelhagen R. (2022). Indoor Navigation Assistance for Visually Impaired People via Dynamic SLAM and Panoptic Segmentation with an RGB-D Sensor. arXiv.

[17] Khoi T.Q., Quang N.A., Hieu N.K. (2021). Object detection for drones on Raspberry Pi potentials and challenges. IOP Conf. Ser. Mater. Sci. Eng..

[18] Lee J., Hwang K.i. (2022). YOLO with adaptive frame control for real-time object detection applications. Multimed. Tools Appl..

[19] Chen Z., Liu X., Kojima M., Huang Q., Arai T. (2021). A wearable navigation device for visually impaired people based on the real-time semantic visual SLAM system. Sensors.

[20] Shaik T.B., Mal R. (2022). Algorithm to Assist Visually Impaired Person for Object Detection in Real Time. Proceedings of the International Conference on Emerging Electronics and Automation.

[21] Kang M., Chae S., Sun J., Yoo J., Ko S. (2015). A novel obstacle detection method based on deformable grid for the visually impaired. IEEE Trans. Consum. Electron..

[22] Kang M., Chae S., Sun J., Lee S., Ko S. (2017). An enhanced obstacle avoidance method for the visually impaired using deformable grid. IEEE Trans. Consum. Electron..

[23] Aladrén A., López-Nicolás G., Puig L., Guerrero J.J. (2016). Navigation assistance for the visually impaired using RGB-D sensor with range expansion. IEEE Syst. J..

[24] Lin S., Wang K., Yang K., Cheng R. KrNet: A kinetic real-time convolutional neural network for navigational assistance. Proceedings of the International Conference on Computers Helping People with Special Needs.

[25] Parikh N., Shah I., Vahora S. Android smartphone based visual object recognition for visually impaired using deep learning. Proceedings of the 2018 International Conference on Communication and Signal Processing (ICCSP).

[26] Tapu R., Mocanu B., Bursuc A., Zaharia T. A smartphone-based obstacle detection and classification system for assisting visually impaired people. Proceedings of the IEEE International Conference on Computer Vision (ICCV) Workshops.

[27] Badrloo S., Varshosaz M., Pirasteh S., Li J. (2022). Image-based obstacle detection methods for the safe navigation of unmanned vehicles: A review. Remote Sens..

[28] Dong X., Garratt M.A., Anavatti S.G., Abbass H.A. (2022). Towards real-time monocular depth estimation for robotics: A survey. IEEE Trans. Intell. Transp. Syst..

[29] Zereen A.N., Corraya S. Detecting real time object along with the moving direction for visually impaired people. Proceedings of the 2016 2nd International Conference on Electrical, Computer Telecommunication Engineering (ICECTE).

[30] Vaidya S., Shah N., Shah N., Shankarmani R. Real-time object detection for visually challenged people. Proceedings of the 2020 4th International Conference on Intelligent Computing and Control Systems (ICICCS).

[31] Shadi S., Hadi S., Nazari M.A., Hardt W. Outdoor navigation for visually impaired based on deep learning. Proceedings of the CEUR Workshop Proceedinds.

[32] Kumar S., Mishra D.N., Ganie S.M., Bharathikannan R., Vijayakanthan K. (2023). Artificial Intelligence Solutions for the Visually Impaired: A Review. Handbook of Research on AI and Knowledge Engineering for Real-Time Business Intelligence.

[33] Rana L., Rehman A.U., Javaid S., Ali T.M. (2022). A Novel Model-Driven Approach for Visual Impaired People Assistance OPTIC ALLY. Proceedings of the 2022 Third International Conference on Latest trends in Electrical Engineering and Computing Technologies (INTELLECT).

[34] Saxena A., Schulte J., Ng A.Y. Depth Estimation Using Monocular and Stereo Cues. Proceedings of the IJCAI.

[35] Duman S., Elewi A., Yetgin Z. (2019). Design and implementation of an embedded real-time system for guiding visually impaired individuals. Proceedings of the 2019 International Artificial Intelligence and Data Processing Symposium (IDAP).

[36] Sohan M., Sai Ram T., Reddy R., Venkata C. (2024). A Review on YOLOv8 and Its Advancements. Proceedings of the International Conference on Data Intelligence and Cognitive Informatics.

[37] Jocher G., Chaurasia A., Qiu J. (2023). YOLO by Ultralytics.

[38] Xiao X., Feng X. (2023). Multi-object pedestrian tracking using improved YOLOv8 and OC-SORT. Sensors.

[39] Wojke N., Bewley A., Paulus D. Simple online and realtime tracking with a deep association metric. Proceedings of the 2017 IEEE International Conference on Image Processing (ICIP).

[40] Rublee E., Rabaud V., Konolige K., Bradski G. ORB: An efficient alternative to SIFT or SURF. Proceedings of the 2011 International Conference on Computer Vision.

[41] Muhammad A., Zalizniak V. (2011). Practical Scientific Computing.

[42] Haseeb M.A., Guan J., Ristic-Durrant D., Gräser A. DisNet: A novel method for distance estimation from monocular camera. Proceedings of the 10th Workshop on Planning, Perception and Navigation for Intelligent Vehicles (PPNIV18), IROS.

[43] Redmon J., Farhadi A. (2018). YOLOv3: An incremental improvement. arXiv.

[44] Trucco E., Verri A. (1998). Introductory Techniques for 3-D Computer Vision.

[45] Hartley R., Zisserman A. (2004). Multiple View Geometry in Computer Vision.

[46] Fiorini P., Shiller Z. (1998). Motion planning in dynamic environments using velocity obstacles. Int. J. Robot. Res..

[47] Clark-Carter D.D., Heyes A.D., Howarth C.I. (1986). The efficiency and walking speed of visually impaired people. Ergonomics.

[48] Robineau D., Baden P., Dhani A., Dark M., Bhagat A., Mann H. (2018). Reported Road Casualties Great Britain: 2017.

[49] Walz F., Hoefliger M., Fehlmann W. (1983). Speed Limit Reduction from 60 to 50 km/h and Pedestrian Injuries.

[50] Richards D.C. (2010). Relationship between Speed and Risk of Fatal Injury: Pedestrians and Car Occupants.

[51] Rebollo-Soria M.C., Arregui-Dalmases C., Sánchez-Molina D., Velázquez-Ameijide J., Galtés I. (2016). Injury pattern in lethal motorbikes-pedestrian collisions, in the area of Barcelona, Spain. J. Forensic Leg. Med..

[52] Short A., Grzebieta R., Arndt N. (2007). Estimating bicyclist into pedestrian collision speed. Int. J. Crashworth..

[53] Chandra S., Bharti A.K. (2013). Speed distribution curves for pedestrians during walking and crossing. Procedia Soc. Behav. Sci..

[54] Freer C. (2019). Vehicle Speed Compliance Statistics, Great Britain: 2018.

[55] Nie B., Li Q., Gan S., Xing B., Huang Y., Li S.E. (2021). Safety envelope of pedestrians upon motor vehicle conflicts identified via active avoidance behaviour. Sci. Rep..

[56] Bernardin K., Stiefelhagen R. (2008). Evaluating multiple object tracking performance: The clear mot metrics. EURASIP J. Image Video Process..

[57] Lin T., Maire M., Belongie S., Hays J., Perona P., Ramanan D., Dollár P., Zitnick C.L. Microsoft COCO: Common objects in context. Proceedings of the European Conference on Computer Vision.

[58] Shao S., Zhao Z., Li B., Xiao T., Yu G., Zhang X., Sun J. (2018). Crowdhuman: A benchmark for detecting human in a crowd. arXiv.

[59] Wen L., Du D., Cai Z., Lei Z., Chang M.C., Qi H., Lim J., Yang M.H., Lyu S. (2020). UA-DETRAC: A new benchmark and protocol for multi-object detection and tracking. Comput. Vis. Image Underst..

[60] Milan A., Leal-Taixé L., Reid I., Roth S., Schindler K. (2016). MOT16: A benchmark for multi-object tracking. arXiv.

[61] Zhao Y., Yan C., Wang Q. (2022). CPU tracking algorithm for lightweight vehicles based on deepsort. Proceedings of the 2022 18th International Conference on Computational Intelligence and Security (CIS).

[62] Held D., Thrun S., Savarese S. (2016). Learning to track at 100 fps with deep regression networks. Proceedings of the Computer Vision—ECCV 2016: 14th European Conference.

[63] Zagoruyko S., Komodakis N. Learning to compare image patches via convolutional neural networks. Proceedings of the IEEE Conference on computer Vision and Pattern Recognition.

[64] Yazdi M., Bouwmans T. (2018). New trends on moving object detection in video images captured by a moving camera: A survey. Comput. Sci. Rev..

[65] Zhang H., Zhang J., Wu Q., Qian X., Zhou T., Fu H. (2017). Extended kernel correlation filter for abrupt motion tracking. KSII Trans. Internet Inf. Syst..

[66] Kuen J., Lim K.M., Lee C.P. (2015). Self-taught learning of a deep invariant representation for visual tracking via temporal slowness principle. Pattern Recognit..

[67] Howard A.G., Zhu M., Chen B., Kalenichenko D., Wang W., Weyand T., Andreetto M., Adam H. (2017). Mobilenets: Efficient convolutional neural networks for mobile vision applications. arXiv.

[68] Meers S., Ward K. A substitute vision system for providing 3D perception and GPS navigation via electro-tactile stimulation. Proceedings of the International Conference on Sensing Technology.

[69] Tao M., Li X., Xie R., Ding K. (2023). Pedestrian Identification and Tracking within Adaptive Collaboration Edge Computing. Proceedings of the 2023 26th International Conference on Computer Supported Cooperative Work in Design (CSCWD).

